# Human CD46 humanized mice reveal interferon associated pulmonary immune remodeling during human adenovirus type 7 lung infection

**DOI:** 10.3389/fimmu.2026.1919716

**Published:** 2026-10-13

**Authors:** Zhongying Yang, Zijun Meng, Mingling Wan, Ying Lu, Lifen Rao, Hui Qu, Jianhua Wei, Jing Liao, Enmei Liu, Na Zang

**Affiliations:** 1 Department of Respiratory Medicine, Children’s Hospital of Chongqing Medical University, Chongqing, China; 2 National Clinical Research Center for Children and Adolescents' Health and Diseases, Children's Hospital of Chongqing Medical University, Chongqing, China; 3 Ministry of Education Key Laboratory of Child Development and Disorders, Children's Hospital of Chongqing Medical University, Chongqing, China; 4 Chongqing Key Laboratory of Child Rare Diseases in Infection and Immunity, Children's Hospital of Chongqing Medical University, Chongqing, China

**Keywords:** human adenovirus type 7, humanized mouse, interferon response, pediatric pneumonia, single-cell RNA sequencing

## Abstract

**Background:**

Human adenovirus type 7(HAdV-7) is a major cause of severe pediatric pneumonia, but the mechanisms linking viral infection to pulmonary immune remodeling and lung pathology remain incompletely understood. Progress has been limited by the lack of physiologically relevant animal models that recapitulate human susceptibility.

**Methods:**

We generated human CD46 (hCD46) humanized mice and evaluated pulmonary viral burden, recoverable infectious virus, and lung pathology after HAdV-7 infection. Single-cell RNA sequencing was performed on lung tissues from wild type and hCD46 humanized mice under control and infected conditions to characterize cellular composition, transcriptional states, and intercellular communication. Human PBMC bulk transcriptomes from pediatric HAdV infection were further analyzed to assess the systemic relevance of mouse lung derived immune programs.

**Results:**

hCD46 humanized mice showed increased pulmonary viral burden, recoverable infectious virions, and more severe lung pathology after HAdV-7 infection. Single-cell profiling revealed depletion of resident alveolar macrophages, pronounced expansion of inflammatory macrophage subsets, and remodeling of lymphoid transcriptional states in infected hCD46 lungs. These changes were accompanied by prominent type I/II interferon and interferon associated chemokine programs. B cells and CD4^+^ and CD8^+^ T cells showed prominent interferon stimulated genes (ISGs) associated transcriptional states, together with alterations in selected effector- and memory-associated programs and computationally inferred trajectories. Projection of mouse lung derived signatures onto transcriptomes of human peripheral blood mononuclear cell showed that selected interferon associated myeloid and lymphoid programs were also detectable systemically in pediatric HAdV infection.

**Conclusions:**

HAdV-7 infection in hCD46 humanized mice was associated with an interferon enriched pulmonary inflammatory environment, prominent inflammatory myeloid remodeling, and altered lymphoid transcriptional states. This susceptible mouse model provides a useful experimental tool for studying HAdV-7 associated lung disease and for preclinical evaluation of antiviral, immunomodulatory, and vaccine strategies.

## Introduction

Human adenoviruses (HAdVs) are important respiratory pathogens.Children and immunocompromised individuals are particularly vulnerable to severe adenoviral pneumonia, which can progress to respiratory failure, acute respiratory distress syndrome, and central nervous system dysfunction (1–3). Some pediatric patients further develop chronic pulmonary sequelae such as bronchiolitis obliterans, causing substantial long term morbidity (4, 5). Among circulating serotypes, human adenovirus type 7 (HAdV-7) is a major cause of pediatric adenoviral infection and is associated with more severe disease and higher mortality than many other serotypes (6–8). Studies have reported multiple outbreaks and epidemics of HAdV-7 worldwide (9, 10). Therefore, elucidating the pathogenic mechanisms of severe HAdV-7 pneumonia is of major importance.

Membrane cofactor protein (MCP/CD46) is a type I transmembrane glycoprotein broadly expressed on human cells except erythrocytes. In addition to regulating complement activity, CD46 serves as a receptor for several human specific pathogens, including group A Streptococcus, pathogenic Neisseria, measles virus, human herpesviruses, and certain adenovirus serotypes such as HAdV-7 (11). Experimental animal models are indispensable for studying infectious disease pathogenesis. Human CD46 (hCD46) transgenic mice have been widely used in studies of measles (12, 13)and human herpesvirus infections (14), streptococcal infections (15), oncolytic virus evaluation (16) and gene therapy applications (17, 18). Several animal models have been used to study HAdV infection, although each captures only selected aspects of human disease. Conventional rodents, including mice, cotton rats, and Syrian hamsters, are experimentally tractable but are limited by HAdV type-dependent permissiveness and species-specific host restrictions, whereas tree shrews can support productive infection by species B HAdVs but have fewer available immunological and genetic tools (19, 20). Human receptor-expressing mouse models, including hDSG2, hCD46/hDSG2, and hCAR/hCD46/hDSG2 mice, have improved the study of HAdV-B receptor usage, pathogenesis, and therapeutic efficacy *in vivo (*21–23). Nevertheless, pulmonary immune remodeling following HAdV-7 infection in receptor-humanized mice remains incompletely characterized at single-cell resolution.

Here, we use an hCD46 humanized mouse model to examine HAdV-7 associated lung disease and the accompanying pulmonary immune changes at single-cell resolution. By combining virological, pathological, and scRNA-seq analyses, we evaluated viral susceptibility, lung injury, and immune cell remodeling in this model, providing additional information for future pathogenesis and intervention studies of HAdV-7.

## Methods

### Virus

The HAdV-7 strain(CQ45_2019, MT113943) used in this study was originally isolated from the nasopharyngeal aspirates of a patient infected with HAdV-7. Viral particles were purified via standard cesium chloride gradient centrifugation (24), and the median tissue culture infectious dose(TCID50) was determined as previously described (25).

### Animals, experimental design, and HAdV-7 challenge

The ROSA26-hCD46 knock-in mouse model on a C57BL/6 background was generated by Cyagen Biosciences (Suzhou, China) using CRISPR/Cas9 technology. Briefly, a donor vector containing a CAG-human CD46 CDS-polyA expression cassette was constructed, and the entire cassette was inserted into intron 1 of the Gt(ROSA)26Sor locus. Cas9 protein, sgRNA, and the donor vector were co-injected into fertilized mouse oocytes, which were subsequently transferred into pseudopregnant recipient females to generate F0 founder mice. Positive F0 founders were crossed with C57BL/6 mice to obtain heterozygous F1 ROSA26-hCD46^KI/+^ offspring, and F1 heterozygous mice were subsequently intercrossed to generate homozygous F2 ROSA26-hCD46KI/KI mice. Homozygous ROSA26-hCD46KI/KI mice were used throughout the present study. Because the availability of homozygous knock-in mice was limited, both male and female mice aged 6–8 weeks with comparable body weights were included without prespecified sex restriction. Eligible mice were randomly assigned to four groups: WT_Control, WT_HAdV-7, hCD46_Control, and hCD46_HAdV-7. No formal *a priori* statistical power calculation was performed; sample sizes were determined based on the availability of age- and body-weight-matched homozygous animals and experimental feasibility. The exact number of animals used for each experimental endpoint is reported in the corresponding figure legends. For the scRNA-seq experiment, three independent mice per group were analyzed, with each mouse processed as an independent 10x Genomics library and no sample pooling. Before intranasal inoculation, mice were weighed and anesthetized by intraperitoneal administration of 10% chloral hydrate at 3–5 μL/g body weight. Mice in the infected groups received a total of 80 μL of HAdV-7 working suspension intranasally, administered as four sequential 20-μL aliquots. The total delivered infectious dose was 4.20 × 10^7^ TCID_50_ per mouse. Sterile PBS was used as the vehicle for the viral inoculum, and control mice received an equal total volume of 80 μL sterile PBS using the same administration procedure. Body weight was monitored daily following inoculation. At 5 days post infection(dpi), mice were exsanguinated by orbital bleeding and subjected to transcardial pulmonary perfusion before lung collection. After opening the thoracic cavity, a small incision was made in the left atrium, and 10 mL of cold PBS was perfused through the cardiac apex until the lungs became uniformly pale. Perfused lung tissues were subsequently collected for viral-load determination, histopathological analysis, scRNA-seq, and assessment of recoverable infectious virus. For the latter, lung homogenates from HAdV-7-infected WT and hCD46 mice were inoculated onto A549 cells, and cytopathic effects (CPE) were evaluated qualitatively by light microscopy to compare recoverable viral infectivity between the two infected groups.All animal experiments were performed in a specific pathogen free (SPF) facility at the Experimental Animal Center of Cyagen Biosciences, Inc and were approved by the Institutional Animal Care and Use Committee (IACUC) [Approval No. IACUC-(Provisional)-SQ2023AAA032266-01].

### Western blot

Lung tissues were lysed using the Total Protein Extraction Kit (KeyGEN BioTECH), and lysates were boiled for 10 min. Protein concentrations were measured using the Pierce BCA Protein Assay Kit. Equal amounts of protein were separated by SDS-PAGE and transferred to nitrocellulose membranes. After blocking with 5% skim milk in PBS for 1 h at room temperature, membranes were incubated overnight at 4 °C with anti-CD46 antibody (ET7107-94, HUABIO, 1:1000), followed by HRP-conjugated goat anti-rabbit IgG (SA00001-2, Proteintech, 1:5000) for 1 h at room temperature. Bands were visualized using a ChemiDoc chemiluminescence imaging system.

### DNA extraction from lung tissue and viral copy number quantification

Viral DNA was extracted from lung tissue using the QIAamp MinElute Virus Spin Kit (Qiagen). Quantitative real time PCR was performed using Taq Pro Universal SYBR qPCR Master Mix (Vazyme) with primers targeting the adenoviral hexon gene, the primer sequences are listed in **Supplementary Table S1**. A pMD19-T plasmid containing the adenoviral hexon gene (TAKARA), provided by Professor Zhang Qiwei, was used as the standard for viral load quantification.

### Hematoxylin and eosin staining, immunohistochemistry, and histopathological assessment

Left lungs were fixed in 4% paraformaldehyde for >24 h, dehydrated, embedded in paraffin, and sectioned at 4 μm. Sections were stained with Harris hematoxylin for 5–8 min, differentiated in 1% hydrochloric acid ethanol, blued in 0.6% ammonia water, counterstained with eosin for 3 min, and mounted with neutral resin. For hCD46 immunohistochemistry, sections underwent citrate-based antigen retrieval, followed by blocking with 3% H_2_O_2_ for endogenous peroxidase and with 5% BSA for blocking, and overnight incubation with a CD46 Rabbit mAb (Zenbio; 1:100), followed by secondary antibody incubation, DAB development, hematoxylin counterstaining, and whole slide scanning. Histopathological lesions were independently evaluated by two investigators blinded to group allocation using a previously described semi-quantitative histopathological score (HPS) (26), and alveolar septal thickness was quantified in QuPath from three randomly selected parenchymal fields per mouse, with eight septa measured per field and averaged for statistical analysis (27).

### ScRNA-seq library preparation, data processing, and quality control

Single-cell libraries were prepared using the 10x Genomics Chromium Single Cell 3′ Library & Gel Bead Kit v2 according to the manufacturer’s instructions (Ou Yi Bio, Shanghai). Briefly, cells were encapsulated into Gel Bead-in-Emulsions (GEMs), reverse-transcribed, and barcoded cDNA was amplified by PCR. Libraries were generated after fragmentation, end repair, adaptor ligation, and index PCR, quantified by Qubit, and sequenced on an Illumina HiSeq 4000 platform with 150-bp paired-end reads.

Raw data were processed using Cell Ranger (v9.0.0). Reads were aligned to the mouse reference genome (mm10). The scRNA-seq dataset comprised 12 independent libraries from 12 individual mice (n = 3 per group), with one library generated per mouse and no sample pooling. Downstream analyses were performed in Seurat (v5.1.0). Cell doublets were identified independently within each sample using scDblFinder and excluded. Cells with 800–10, 000 detected genes, 1, 000–40, 000 UMIs, mitochondrial fraction <10%, and ribosomal fraction <35% were retained. Ambient RNA contamination was estimated using decontX with sample identity as the batch variable and preliminary cluster information, and cells with estimated contamination ≥20% were excluded; uncorrected count matrices were retained for downstream analysis. After quality control, doublet, and ambient-RNA filtering, 49, 113 cells were retained from an initial merged dataset of 73, 336 cells, corresponding to an overall exclusion rate of approximately 33%. Across the 12 libraries, the mean sequencing depth was approximately 68, 100 reads per cell (range, 40, 231–147, 292). The median numbers of detected genes and UMIs per cell were approximately 2, 366 and 8, 552, respectively, and mean sequencing saturation was approximately 70%.

### Dimensionality reduction, clustering, and integrated data analysis

Expression data were log-normalized using Seurat LogNormalize with a scale factor of 10, 000, and 2, 500 highly variable genes were selected. Cell-cycle scores were regressed during scaling, followed by principal component analysis and Harmony batch correction using sample identity as the batch variable. The first 12 Harmony-corrected dimensions were used for shared-nearest-neighbor graph construction and UMAP visualization, and Louvain clustering was performed at a resolution of 0.4. Cell types were annotated based on canonical markers and published single-cell references for lung and immune populations (28–30). Cluster marker genes were identified using the Wilcoxon rank-sum test in Seurat, with a minimum detection fraction of 25% and a log_2_ fold change threshold of 0.25. Cell population abundance was analyzed at the individual mouse level using logit-transformed proportions with Benjamini–Hochberg correction. Condition level differential expression analysis was performed using DESeq2 (v1.46.0) after aggregating raw counts by mouse within each major cellular compartment. Functional enrichment analyses were performed using GO and KEGG databases, and signature scores were calculated for interferon stimulated genes (ISGs) and inflammatory programs. Differentiation trajectories were reconstructed using Monocle3 and ordered in pseudotime. Potential ligand–receptor communication patterns were inferred using CellChat, including interaction number, interaction strength, pathway-level information flow, and sender-receiver centrality analyses.

### Flow cytometry and reverse transcription quantitative real-time PCR validation

At 5 dpi, perfused lung tissues were processed into single-cell suspensions. For flow cytometry, viable CD45^^+^^ leukocytes from WT_Control (n = 4), WT_HAdV-7 (n = 5), hCD46_Control (n = 5), and hCD46_HAdV-7 (n = 5) mice were analyzed. T cells were identified as CD3^^+^^ cells and further analyzed for CD4 and CD8 expression. CD4^^+^^ central memory-like (Tcm-like) and CD8^^+^^ effector memory-like (Tem-like) cells were defined as CD44^high^CD62L^high^ and CD44^high^CD62L^low^, respectively. B cells were identified as CD19^^+^^ cells, with CD138^^+^^ plasma-like and MHC-II^^+^^ populations further quantified. Data were analyzed using FlowJo, and frequencies were calculated relative to the indicated parent populations.Lung macrophages were magnetically enriched from perfused lung single-cell suspensions of hCD46 control and HAdV-7 infected mice at 5 dpi. Total RNA was extracted and reverse-transcribed into cDNA, followed by quantitative real-time PCR for Cxcl10, Cxcl11, Isg15, Ifit1, Irf7, and Stat1. Gapdh was used as the internal reference, and relative mRNA expression was calculated using the 2^−ΔΔCt^ method. Primer sequences are provided in **Supplementary Table S1**.

### Peripheral blood mononuclear cell and clinical data collection

Peripheral blood samples were collected within 24 h after admission from pediatric patients with HAdV associated respiratory infection and from healthy children undergoing routine outpatient health examinations. HAdV associated respiratory infection was diagnosed according to the Chinese guideline for the diagnosis and treatment of adenovirus pneumonia in children, based on compatible clinical manifestations, radiological evidence of pneumonia, HAdV positive PCR results from nasopharyngeal aspirates, physician recorded diagnosis, and the absence of another pathogen judged as the primary cause of disease (31). Viral serotyping was not performed in this pediatric cohort; therefore, the human transcriptomic analysis was interpreted as reflecting HAdV associated immune responses rather than HAdV-7 specific responses.Disease severity was classified according to the Guideline for the Diagnosis and Treatment of Pediatric Community Acquired Pneumonia of China (32). Healthy controls were outpatient children without acute infectious symptoms within the previous two weeks and without known underlying diseases. Patients receiving immunosuppressive therapy or lacking essential clinical information were excluded. Clinical data were collected from standardized medical records. Laboratory indicators included white blood cell(WBC), platelet count, red blood cell(RBC), hemoglobin, lymphocyte count, lymphocyte percentage, neutrophil count, neutrophil percentage, total protein, albumin, globulin, lactate dehydrogenase(LDH), alanine aminotransferase(ALT), and aspartate aminotransferase(AST). This study was reviewed and approved by the Ethics Committee of the Children’s Hospital of Chongqing Medical University (approval number: 2024-217). Because anonymized residual samples from routine clinical testing stored in the institutional biobank were used, the requirement for informed consent was waived by the ethics committee.

### PBMC bulk RNA-seq processing and analysis

Total RNA was extracted from PBMC samples using TRIzol reagent according to the manufacturer’s instructions. Poly(A)^+^ RNA was enriched from 1 μg total RNA using Dynabeads Oligo(dT), fragmented, reverse transcribed, and used for strand specific library construction. After adaptor ligation and PCR amplification, libraries with an average insert size of approximately 300 ± 50 bp were sequenced on an Illumina NovaSeq 6000 platform using paired-end 150-bp sequencing. Raw gene level count matrices were used for downstream analysis, and duplicate gene symbols were collapsed by summing raw counts. Samples were matched to clinical metadata and assigned to the HAdV or Control group. Differential expression analysis was performed using DESeq2. Genes with counts ≥10 in at least three samples were retained, and variance stabilizing transformation was applied for principal component analysis and visualization. Differentially expressed genes between HAdV infected patients and controls were identified using the DESeq2 Wald test. For volcano plot visualization, genes with adjusted P value <0.05 and absolute log2 fold change >1 were considered significant, and selected interferon stimulated, myeloid inflammatory, and lymphoid associated genes were labeled only when they met this threshold.

Hallmark gene set enrichment analysis was performed using the DESeq2 Wald statistic from the HAdV-versus-Control comparison as the preranked metric. Hallmark gene sets were obtained using msigdbr, and enrichment analysis was conducted with fgseaMultilevel. Enriched pathways were summarized by normalized enrichment score and adjusted P value. Mouse lung scRNA-seq derived signatures were generated from the top 20 marker genes of selected macrophage and lymphoid clusters, including Macro_Cxcl10-like, Macro_Gpnmb-like, Macro_C1qa-like, CD4_ISG, CD8_ISG, B_ISG, integrated lymphoid ISG, B activation/memory, CD8 effector/memory, and CD4 memory/effector programs. Mouse gene symbols were matched to the human PBMC expression matrix using uppercase gene symbol matching, and only detected genes were retained. For each signature, sample level scores were calculated as the mean z-score of detected signature genes across samples. Signature scores were compared between HAdV and Control groups using two sided Wilcoxon rank-sum tests with Benjamini Hochberg correction. Signature level concordance was assessed by calculating the mean log2 fold change of genes within each signature in the HAdV-versus-Control DESeq2 results, and one sample Wilcoxon tests with Benjamini Hochberg correction were used to determine whether signature gene log2 fold changes were directionally shifted from zero. Core gene heatmaps were generated from variance stabilized expression values after gene wise z-score scaling, with up to five detected genes displayed per signature in compact heatmaps. Exploratory associations between scRNA derived signature scores and laboratory indicators were assessed using Spearman correlation and visualized as correlation heatmaps. Stars indicate nominal Spearman P values for exploratory visualization only.

### Statistical analysis

Statistical analyses and visualization were performed using GraphPad Prism (v10.2.3) and R (v4.3.1). Data presentation and statistical tests are described in the corresponding figure legends and method sections. For animal experiments and other normally distributed quantitative measurements, data are presented as mean ± SEM. Pulmonary viral loads were compared using a two tailed exact Mann–Whitney U test, and RT-qPCR data, were performed using unpaired two tailed Student’s t tests when variances were equal; otherwise, Welch’s t tests were applied. Longitudinal body-weight data were analyzed using two-way repeated-measures ANOVA with group and time as factors and Geisser–Greenhouse correction, followed by Tukey’s multiple-comparisons test. Histopathological scores were analyzed using the Kruskal–Wallis test followed by Dunn’s multiple-comparisons test. Alveolar septal thickness and flow cytometric data were analyzed using ordinary one-way ANOVA followed by Tukey’s multiple-comparisons test when variances were equal; when the Brown–Forsythe test indicated unequal variances (P < 0.05), Brown–Forsythe and Welch ANOVA followed by Dunnett’s T3 multiple-comparisons test was used. For clinical laboratory indicators and PBMC signature scores, two sided Wilcoxon rank sum tests were used. Demographic and clinical characteristics are presented as median (interquartile range [IQR]) or n (%), as appropriate. Age was compared using a two-sided exact Mann–Whitney U test and sex distribution using Fisher’s exact test. Differential expression analysis of PBMC bulk RNA-seq data was performed using DESeq2, with Benjamini Hochberg correction for multiple testing. Hallmark gene set enrichment analysis was performed using fgseaMultilevel. Signature level concordance was assessed using one sample Wilcoxon tests with Benjamini–Hochberg correction. Exploratory associations between scRNA derived signature scores and clinical laboratory indicators were evaluated using Spearman correlation. A P value or adjusted P value <0.05 was considered statistically significant unless otherwise specified.

## Results

### Enhanced HAdV-7 infection severity in hCD46 humanized mice

CD46 is a critical receptor for human adenovirus entry into cells. Using CRISPR/Cas9 gene editing, we successfully inserted the hCD46 gene into C57BL/6 mice. Western blotting and immunohistochemistry consistently demonstrated hCD46 expression in lung tissue, hereas wild type(WT) mice showed no detectable expression (**Figure 1A**). Following intranasal HAdV-7 infection, the viral genome copy number in lung tissue was markedly higher in hCD46 humanized mice than in WT infected mice (P = 0.0286; **Figure 1B**), indicating that hCD46 substantially enhances *in vivo* viral replication. To further confirm productive infection and generation of infectious viral progeny, lung homogenates from infected mice were used to inoculate A549 cells. Homogenates from hCD46 humanized mice induced more pronounced cytopathic effects than those from WT_HAdV-7 mice (**Figure 1C**), consistent with greater recoverable viral infectivity in hCD46 lungs. Body weight trajectories differed significantly among the four groups over time (group × time interaction, P = 0.0008; **Figure 1D**). At 1 dpi, hCD46_HAdV-7 mice showed a significant decrease in body weight compared with hCD46 controls (P = 0.0242). hCD46_HAdV-7 mice maintained lower mean body weights than WT_HAdV-7 mice throughout 1–5 dpi and remained below baseline at 5 dpi. Histopathological analysis revealed inflammatory infiltration, thickening of alveolar walls, epithelial damage, and disruption of alveolar architecture, with more prominent changes observed in hCD46 humanized mice (**Figure 1E**). Histopathological scores also differed significantly among groups (Kruskal–Wallis P = 0.0019), with higher scores in hCD46_HAdV-7 mice than in hCD46 controls (adjusted P = 0.0066; **Figure 1F**). Alveolar septal thickness also differed significantly among the four groups (P = 0.0029) and was greater in hCD46_HAdV-7 mice than in hCD46 controls (mean difference, 3.64 μm; 95% CI, 1.16–6.11 μm; P = 0.0036) and WT_HAdV-7 mice (mean difference, 2.43 μm; 95% CI, 0.11–4.76 μm; P = 0.0388; **Figure 1G**).

**Figure 1:**
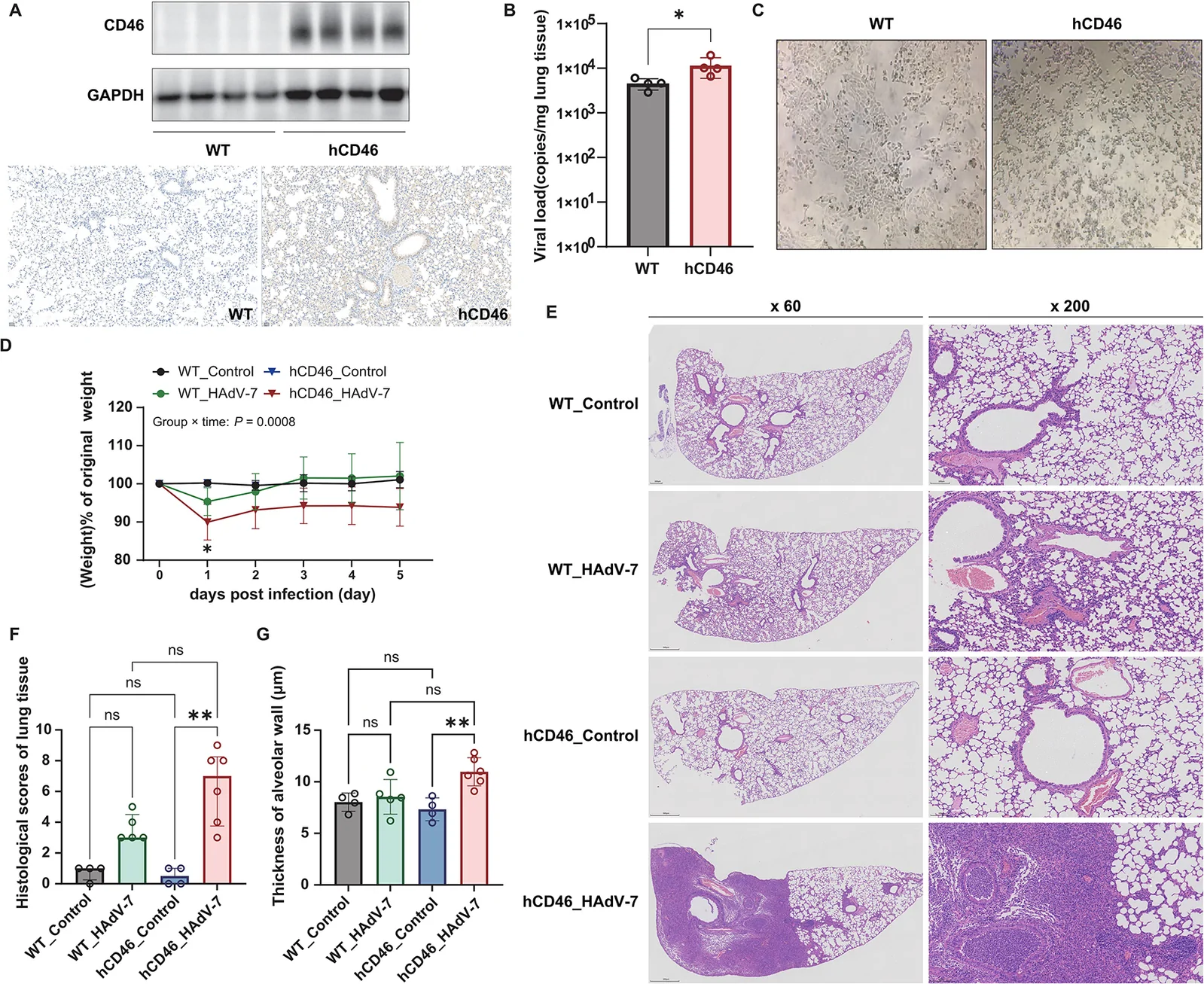
HAdV-7 infection in mice. **(A)** Western blot and immunohistochemical analysis of CD46 expression in lung tissues from WT and hCD46 mice. Original magnification, ×200; scale bar, 25 µm. **(B)** Viral load in lung tissues at 5 dpi of each group. Pulmonary viral loads were compared between WT_HAdV-7 and hCD46_HAdV-7 mice using a two-tailed exact Mann–Whitney U test (n = 4 biologically independent mice per group; P = 0.0286). **(C)** Cytopathic effects observed in A549 cells inoculated with lung homogenates collected from WT_HAdV-7 and hCD46_HAdV-7 mice at 5 dpi. **(D)** Changes in body weight post HAdV-7 infection. Body weight was normalized to the baseline value at day 0. Statistical analysis was performed using two-way repeated measures ANOVA with Geisser–Greenhouse correction followed by Tukey’s multiple-comparisons test; the group × time interaction was significant (P = 0.0008). **(E)** H&E stained lung sections from the indicated groups. Original magnification, ×60 (left) and ×200 (right); scale bars, 500 μm and 100 μm, respectively. **(F)** Blinded semiquantitative histopathological scores of lung tissues. Data are presented as median with interquartile range (IQR), and each dot represents one mouse. Statistical significance was assessed using the Kruskal–Wallis test followed by Dunn’s multiple comparisons test (overall P = 0.0019). **(G)** Quantification of alveolar septal thickness in H&E stained lung sections. Three randomly selected lung parenchymal fields and eight septa per field were analyzed for each mouse, and each dot represents the mean value from one mouse. Data are presented as mean ± SEM. Statistical significance was assessed using ordinary one-way ANOVA followed by Tukey’s multiple-comparisons test (overall P = 0.0029).For animal experiments, n = 4–6 mice per group. *P < 0.05, **P < 0.01; ns, not significant.

### Single-cell atlas of pulmonary cellular remodeling following HAdV-7 infection in hCD46 humanized mice

To define the global impact of HAdV-7 infection on the lung cellular ecosystem, we performed scRNA-seq on lung tissues from WT and hCD46 humanized mice under both steady-state and infected conditions (**Figure 2A**). After quality control, doublet, and ambient RNA filtering, 49, 113 high quality single-cell transcriptomes from 12 independent mice were retained for downstream analysis. Unsupervised clustering and marker-based annotation identified major pulmonary compartments, including immune, epithelial, endothelial, and stromal lineages (**Figure 2B**; **Supplementary Figures S1A–D**) Sample level analysis revealed broad remodeling of pulmonary cell composition following HAdV-7 infection (**Figure 2C**). T cell proportions decreased after infection, reaching statistical significance in WT mice, while a similar downward trend was observed in hCD46 mice. B cell proportions were lower in hCD46_HAdV-7 lungs, whereas plasma cells remained a low frequency population across groups. Neutrophils increased prominently following infection, while the Mac/Mono/DC compartment showed marked expansion in hCD46 mice. In contrast, alveolar macrophages were markedly depleted in both genotypes. Among structural cells, airway epithelial cells and fibroblasts showed decreasing trends after infection, whereas endothelial cells showed a numerical increase in infected hCD46 lungs.

**Figure 2:**
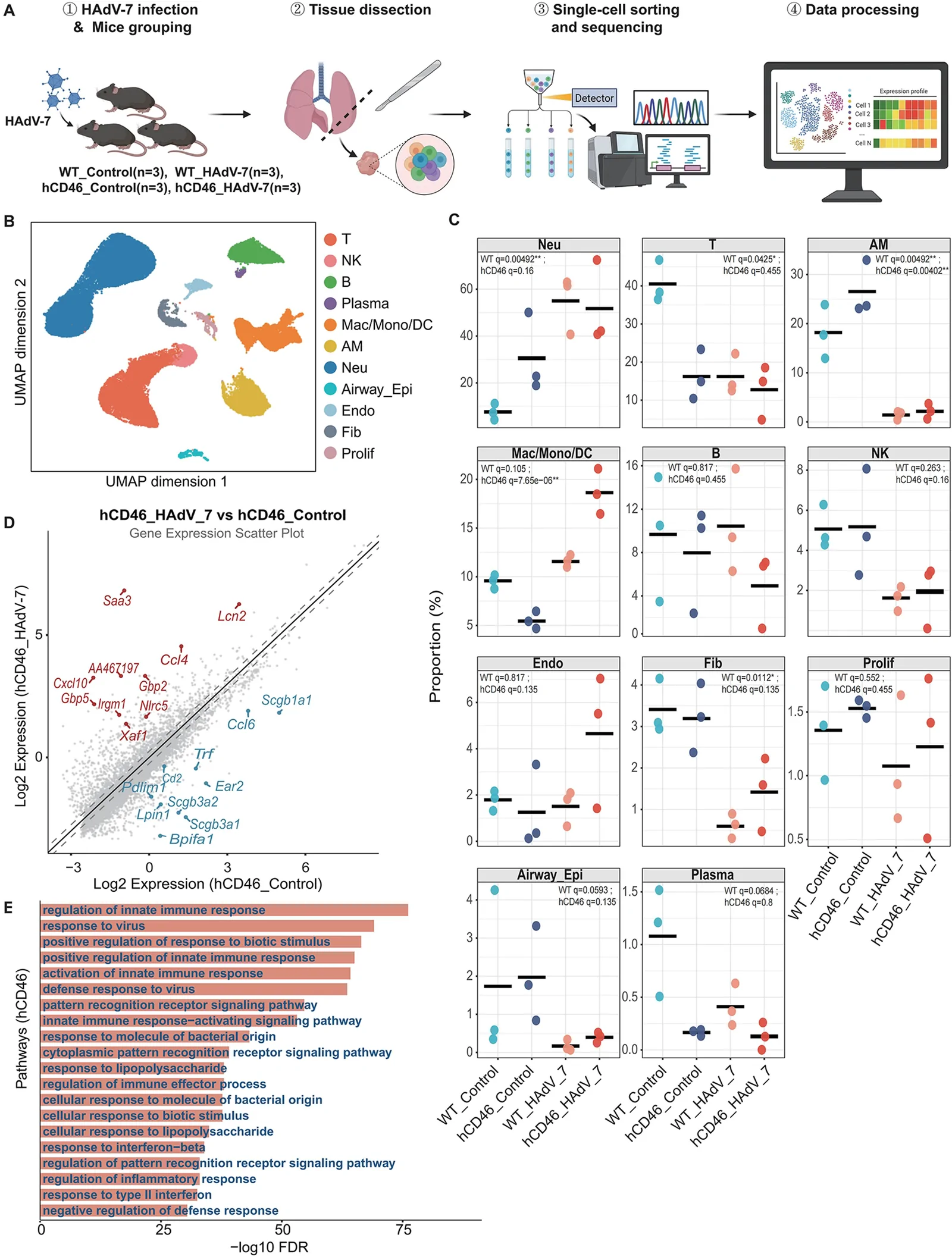
Single-cell atlas of pulmonary cellular remodeling following HAdV-7 infection in hCD46 humanized mice. **(A)** Experimental workflow for single-cell profiling of lung tissues from WT and hCD46 humanized mice under control and HAdV-7-infected conditions (n = 3 biologically independent mice per group). Created with BioRender.com. **(B)** UMAP visualization of major pulmonary cell populations including T cells (T), natural killer cells (NK), B cells **(B)**, plasma cells (Plasma), macrophages, monocytes, and dendritic cells (Mac/Mono/DC), alveolar macrophages (AM), neutrophils (Neu), airway epithelial cells (Airway_Epi), endothelial cells (Endo), fibroblasts (Fib), and proliferating cells (Proli). **(C)** Sample level proportions of major pulmonary cell populations across genotype and infection conditions. Each point represents one individual mouse, and horizontal bars indicate group means. Cell proportions were analyzed using a linear model on logit-transformed proportions, followed by Benjamini–Hochberg correction. q indicates the adjusted P value for infection-versus-control comparisons within each genotype. **(D)** Gene expression scatter plot comparing hCD46_HAdV-7 and hCD46_Control lungs, highlighting representative differentially expressed genes. **(E)** Top 20 enriched pathways in hCD46_HAdV-7 versus hCD46_Control lungs.

At the transcriptional level, WT_HAdV-7 lungs showed increased expression of inflammatory mediators such as S100a8, S100a9, and Il1b, together with enrichment of pattern recognition receptor (PRR) mediated innate immunity, inflammatory responses, and myeloid leukocyte activation (**Supplementary Figures S1E, F**). By contrast, hCD46_HAdV-7 lungs displayed a dominant interferon associated transcriptional signature, including Saa3, Lcn2, Cxcl10, Gbp2, and Irgm1 (**Figure 2D**). Pathway analysis further identified enrichment of type I and type II interferon(IFN-I and IFN-II) responses, in addition to general innate immunity and viral defense (**Figure 2E**). Together, the higher pulmonary viral burden in hCD46 mice was accompanied by a prominent interferon associated transcriptional response and broad remodeling of the pulmonary immune landscape.

### Myeloid remodeling and interferon associated inflammatory transcriptional states following HAdV-7 infection

To delineate myeloid remodeling associated with HAdV-7 infection in hCD46 humanized mice, we performed high resolution profiling of monocytes and macrophages (**Figure 3A**; **Supplementary Figure S2A**). Six major myeloid states were identified, including three monocyte states (Mono_Cd300e, Mono_Idi1, and Mono_Vcan) and three macrophage states (Macro_Cxcl10, Macro_C1qa, and Macro_Gpnmb). Sample level abundance analysis revealed substantial infection associated remodeling of the myeloid compartment (**Figure 3B**). In hCD46 mice, all three monocyte states decreased significantly after infection, whereas Macro_Cxcl10, Macro_C1qa, and Macro_Gpnmb increased significantly. Pathway analysis of the myeloid compartment further revealed that hCD46_HAdV-7 myeloid cells were strongly enriched for type I/II interferon signaling, pattern recognition receptor pathways, and cytokine response programs (**Figure 3C**). In contrast, WT_HAdV-7 myeloid cells showed prominent enrichment of mitochondrial translation and oxidative phosphorylation (**Supplementary Figure S2B**).

**Figure 3:**
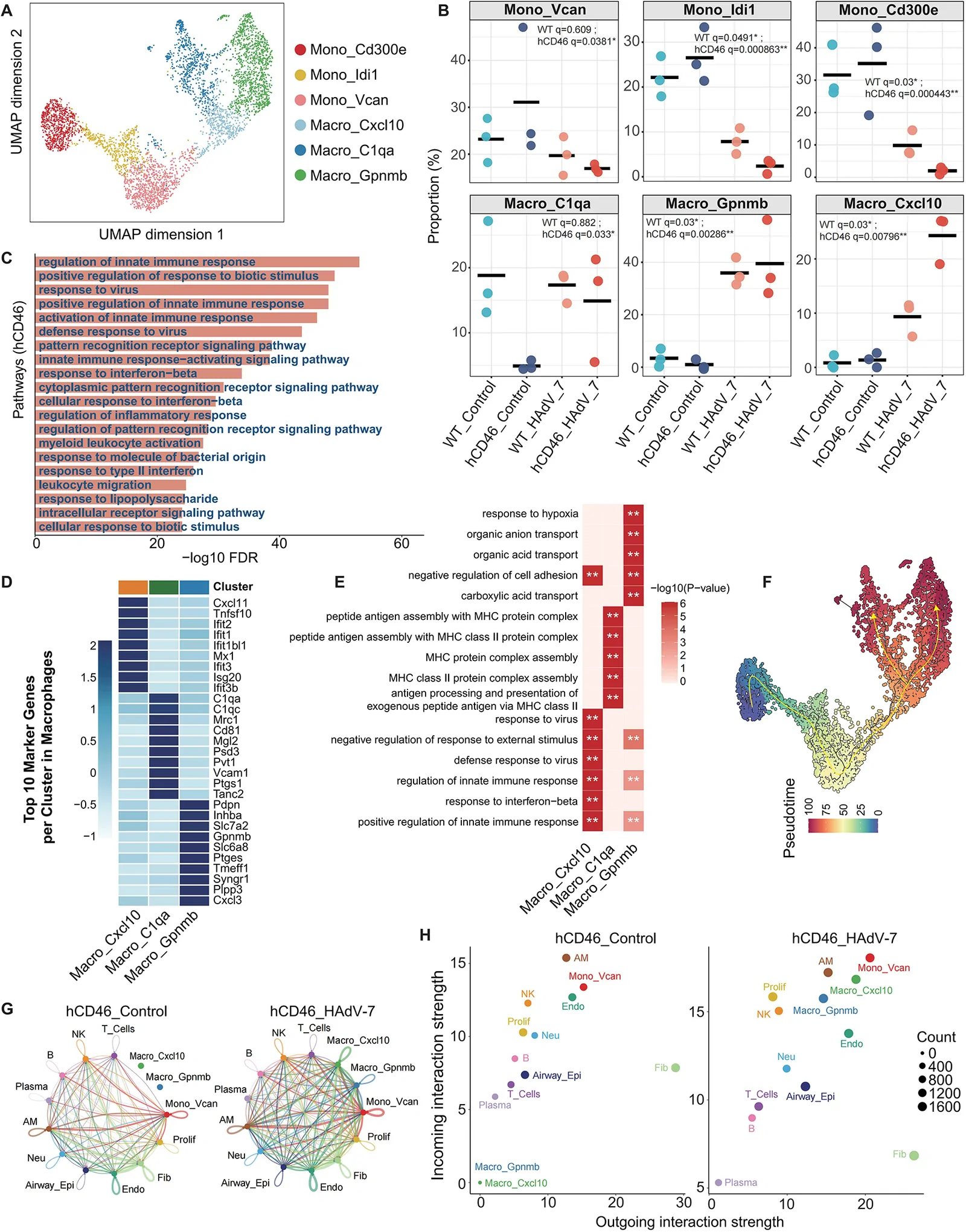
Myeloid remodeling and interferon associated inflammatory transcriptional states following HAdV-7 infection. **(A)** UMAP visualization of monocyte and macrophage populations across all experimental groups. **(B)** Sample level proportions of the indicated myeloid states across experimental groups. Each point represents one biologically independent mouse (n = 3 mice per group), and horizontal bars indicate group means. Cell proportions were analyzed using a linear model on logit-transformed proportions with genotype, infection, and their interaction as predictors, followed by Benjamini–Hochberg correction within the myeloid compartment. q values indicate adjusted P values for infection-versus-control comparisons within each genotype. **(C)** Top 20 enriched pathways in myeloid populations from hCD46_HAdV-7 lungs(hCD46_HAdV-7 versus hCD46_Control). **(D)** Top 10 marker genes defining macrophage subclusters. **(E)** Pathway enrichment analysis of major macrophage subsets. **(F)** Monocle3-inferred monocyte/macrophage trajectory colored by pseudotime. Lines represent computationally inferred relationships among transcriptional states rather than experimentally demonstrated lineage progression. **(G)** CellChat predicted ligand–receptor interaction networks in hCD46_Control and hCD46_HAdV-7 lungs. Edge widths represent inferred interaction strength. **(H)** CellChat signaling role scatter plot(incoming vs outgoing interaction strength) in hCD46 lungs. *q < 0.05; **q < 0.01; ***q < 0.001.

Marker and pathway analyses further distinguished the major macrophage states (**Figures 3D, E**). Macro_Cxcl10 showed a prominent interferon/antiviral and chemokine associated transcriptional profile, characterized by Cxcl10, Cxcl11, Ifit family members, Mx1, Isg20, and other canonical ISGs. Macro_Gpnmb was associated predominantly with hypoxia-, lipid-, and stress-related programs, whereas Macro_C1qa was characterized by antigen presentation and complement associated programs. Consistent with the scRNA-seq findings, RT-qPCR analysis of magnetically enriched pulmonary macrophages independently confirmed significant induction of Cxcl10, Cxcl11, Ifit1, Irf7, Isg15, and Stat1 following HAdV-7 infection in hCD46 mice (all P < 0.05; **Supplementary Figure S2C**). Notably, this interferon associated chemokine signature parallels our previous clinical observations in pediatric patients with severe HAdV-7 pneumonia, where circulating IP-10(CXCL10) and IFN-γ were markedly elevated (33).

Monocle3 analysis further revealed continuous relationships among monocyte and macrophage transcriptional states (**Figure 3F**). Mono_Vcan cells occupied an early region of the inferred trajectory, followed by branches associated with Macro_C1qa- and Macro_Gpnmb-like states. These patterns suggest distinct transcriptional state transitions within the macrophage compartment rather than experimentally demonstrated differentiation routes. Finally, CellChat analysis was used to explore predicted ligand–receptor communication associated with myeloid remodeling. Compared with hCD46_Control lungs, hCD46_HAdV-7 lungs showed broader predicted connectivity involving Macro_Cxcl10, Macro_Gpnmb, and other pulmonary cell populations (**Figure 3G**). CellChat derived signaling scores similarly suggested increased inferred connectivity of selected macrophage populations following infection (**Figure 3H**; **Supplementary Figures S2D, E**).

### Interferon associated remodeling of T cell states and inferred trajectories in hCD46 humanized mice post HAdV-7 infection

CD4^^+^^ T cell subclustering identified heterogeneous transcriptional states across experimental groups (**Figure 4A**; **Supplementary Figure S3A**). Sample level abundance analysis showed no significant infection associated changes in naïve or central memory CD4^^+^^ T cell proportions in hCD46 mice, whereas the CD4^^+^^ T_ISG–Mx1 population showed an increasing trend following infection and the CD4^^+^^ T_Treg–Il1rl1 population increased significantly (**Figure 4B**). Monocle3 analysis identified multiple inferred CD4^^+^^ T cell trajectories, including an ISG associated branch containing CD4^^+^^ T_ISG–Mx1 cells (**Figure 4C**). Consistent with these cellular state changes, pathway enrichment analysis showed antiviral and innate immune pathways, including interferon-β/γ signaling and pattern recognition receptor cascades in hCD46_HAdV-7 CD4^+^ T cells, accompanied by expression of canonical ISGs, including Mx1/2, Ifit1/2/3, Rsad2, Oasl2, Usp18, and Isg15 (**Supplementary Figure S3B**; **Supplementary Table S2**). In contrast, WT_HAdV-7 CD4^^+^^ T cells were enriched for mitochondrial oxidative phosphorylation, respiratory chain assembly, and ATP biosynthesis pathways (**Supplementary Figure S3C**).

**Figure 4:**
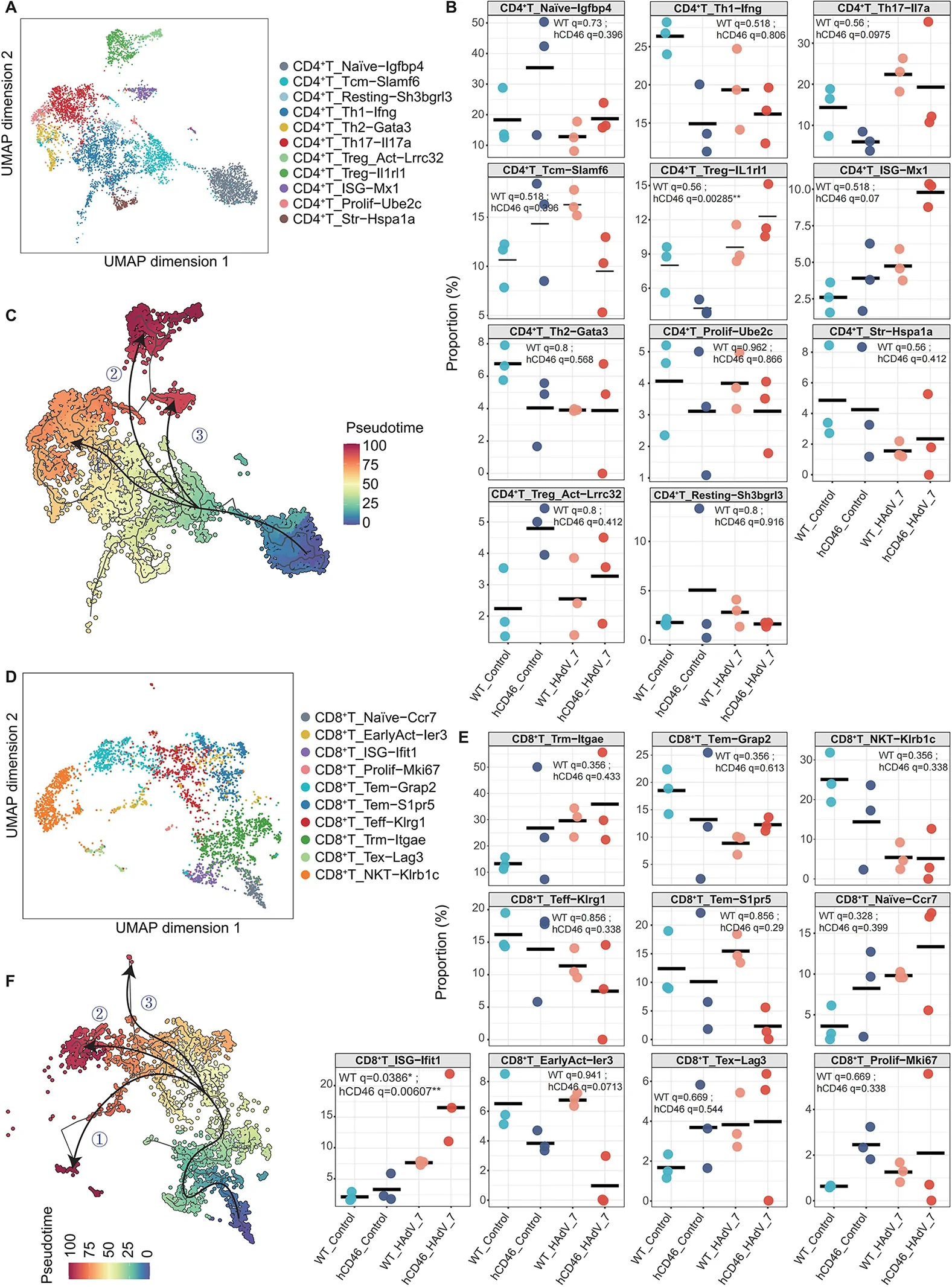
Interferon associated remodeling of T cell states and inferred trajectories in hCD46 humanized mice following HAdV-7 infection. **(A)** UMAP visualization of CD4^^+^^ T cell states across experimental groups, colored by annotated cell states; CD4^^+^^ T cells comprised naïve CD4^^+^^ T cells (CD4^^+^^ T_ Naïve–Igfbp4), central memory CD4^^+^^ T cells (CD4^^+^^ T_Tcm–Slamf6), resting CD4^^+^^ T cells (CD4^^+^^ T_Resting–Sh3bgrl3), T helper 1 cells (CD4^^+^^ T_Th1–Ifng), T helper 2 cells (CD4^^+^^ T_Th2–Gata3), T helper 17 cells (CD4^^+^^ T_Th17–Il17a), activated regulatory T cells (CD4^^+^^ T_Treg_Act–Lrrc32), regulatory T cells (CD4^^+^^ T_Treg–Il1rl1), interferon stimulated CD4^^+^^ T cells (CD4^^+^^ T_ISG–Mx1), proliferating CD4^^+^^ T cells (CD4^^+^^ T_Prolif–Ube2c), and stress response CD4^^+^^ T cells (CD4^^+^^ T_Str–Hspa1a). **(B)** Sample level proportions of CD4^+^ T cell states across genotype and infection conditions. Each point represents one biologically independent mouse (n = 3 mice per group), and horizontal bars indicate group means. Cell proportions were analyzed using a linear model on logit-transformed proportions with genotype, infection, and their interaction as predictors. q values represent Benjamini–Hochberg adjusted P values for infection-versus-control comparisons within each genotype. **(C)** Monocle3 inferred CD4^^+^^ T cell trajectory colored by pseudotime, showing three inferred branches extending from the naïve cell region: the Helper associated branch (path 1), the Regulatory associated branch (path 2), and the Interferon responsive branch (path 3). **(D)** UMAP embedding of CD8^^+^^ T cell states across experimental groups; CD8^^+^^ T cells comprised naïve CD8^^+^^ T cells (CD8^^+^^ T_Naïve–Ccr7), early activated CD8^^+^^ T cells (CD8^^+^^ T_EarlyAct–Ier3), interferon stimulated CD8^^+^^ T cells (CD8^^+^^ T_ISG–Ifit1), proliferating CD8^^+^^ T cells (CD8^^+^^ T_Prolif–Mki67), effector memory CD8^^+^^ T cells (CD8^^+^^ T_Tem–Grap2), circulating effector memory CD8^^+^^ T cells (CD8^^+^^ T_Tem–S1pr5), terminal effector CD8^^+^^ T cells (CD8^^+^^ T_Teff–Klrg1), tissue-resident memory CD8^^+^^ T cells (CD8^^+^^ T_Trm–Itgae), exhausted CD8^^+^^ T cells (CD8^^+^^ T_Tex–Lag3), and NKT-like cells (CD8^^+^^ T_NKT).**(E)** Sample level proportions of CD8^^+^^ T cell states across genotype and infection conditions. Data are displayed and analyzed as described for panel B. **(F)** Monocle3-inferred CD8^^+^^ T cell trajectory colored by pseudotime, showing three inferred branches associated with exhaustion (path 1), NKT/innate-like (path 2), and effector (path 3) transcriptional states.

CD8^^+^^ T cells showed a similar interferon associated transcriptional pattern (**Figure 4D**). Sample level abundance analysis demonstrated a significant increase in the CD8_ISG–Ifit1 population following HAdV-7 infection in both WT and hCD46 mice, whereas the other CD8^^+^^ T cell states showed no significant infection associated changes (**Figure 4E**). Monocle3 analysis identified multiple inferred CD8^^+^^ T cell branches encompassing ISG associated and downstream transcriptional states (**Figure 4F**). Pathway enrichment analysis further showed hCD46_HAdV-7 CD8^^+^^ T cells were predominantly enriched for interferon mediated antiviral and innate immune pathways, together with expression of Irf7, Ifit1/2/3, Rsad2, Mx1/2, Isg15, Oasl2, Gbp6, Usp18, and Cxcl10(**Supplementary Figure S3E**; **Supplementary Table S3**). In comparison, WT_HAdV-7 CD8^^+^^ T cells were enriched predominantly for programs supporting translation, mitochondrial metabolism, and biosynthetic activity (**Supplementary Figure S3F**). Together, these analyses show that T cell remodeling in hCD46_HAdV-7 lungs at 5 dpi was characterized by prominent ISG/antiviral transcriptional programs and altered distributions of T cell states along inferred trajectories. As a complementary phenotypic assessment, flow cytometry showed a significant reduction in total pulmonary CD3^^+^^ T cell frequency following HAdV-7 infection in hCD46 mice, whereas CD4^^+^^ and CD8^^+^^ T cell frequencies and the examined memory-like T cell populations showed no significant infection associated changes (**Supplementary Figure S3G**).

### Interferon associated remodeling of B cell states following HAdV-7 infection in hCD46 humanized mice

B cell subclustering identified eight transcriptional states, including transitional (B_Transitional-Sox4), naïve-like (B_Naïve-like), interferon stimulated (B_ISG-Ifit3), early-memory (B_Mem_Early-Ccr6), core-memory (B_Mem_Core-Cd80), early activated (B_EarlyAct-Junb), proliferating (B_Prolif-Ccnd2), and plasma cell (B_Plasma) populations (**Figure 5A**; **Supplementary Figure S4A**). Sample level abundance analysis revealed distinct infection associated changes in B cell states (**Figure 5B**). B_Mem_Early–Ccr6 increased markedly following infection in WT mice, whereas a comparable increase was not observed in hCD46 mice. In contrast, B_ISG–Ifit3 increased following infection in both genotypes, with a numerically greater increase in hCD46 mice. B_ISG–Ifit3 cells expressed a prominent interferon associated gene program(e.g., Rtp4, Ifit3, Irf7, Oas/Oasl family members, and Usp18) (**Supplementary Table S4**). Flow cytometry at 5 dpi showed no significant infection associated changes in total CD19^^+^^ B cells, CD19^^+^^CD138^+^ plasma-like cells, or MHC-II^^+^^ B cell frequencies (**Supplementary Figure S4B**). Functional enrichment analysis further highlighted distinct B cell transcriptional programs in WT and hCD46 mice. WT B cells were enriched for pathways associated with protein synthesis, cellular metabolism, and MHC class II mediated antigen presentation, whereas hCD46 B cells preferentially engaged virus response, interferon-β/γ signaling, pattern recognition receptor pathways, and innate immune activation programs (**Figure 5C**; **Supplementary Figure S4C**). Pseudotime analysis placed B_ISG–Ifit3 cells within the mid-to-late region of the inferred B cell trajectory in hCD46_HAdV-7 lungs (**Supplementary Figure S4D**). CellChat analysis further predicted altered ligand–receptor connectivity involving B cell subsets following infection. In hCD46_HAdV-7 lungs, B_Plasma and B_Prolif–Ccnd2 showed lower predicted connectivity with multiple pulmonary cell populations compared with hCD46_Control lungs (**Figure 5D**; **Supplementary Figure S4E**). B cell subsets generally showed relatively greater inferred incoming than outgoing signaling scores, including the B_ISG–Ifit3 population (**Figure 5E**). These findings indicate interferon associated remodeling of pulmonary B cell states following HAdV-7 infection.

**Figure 5:**
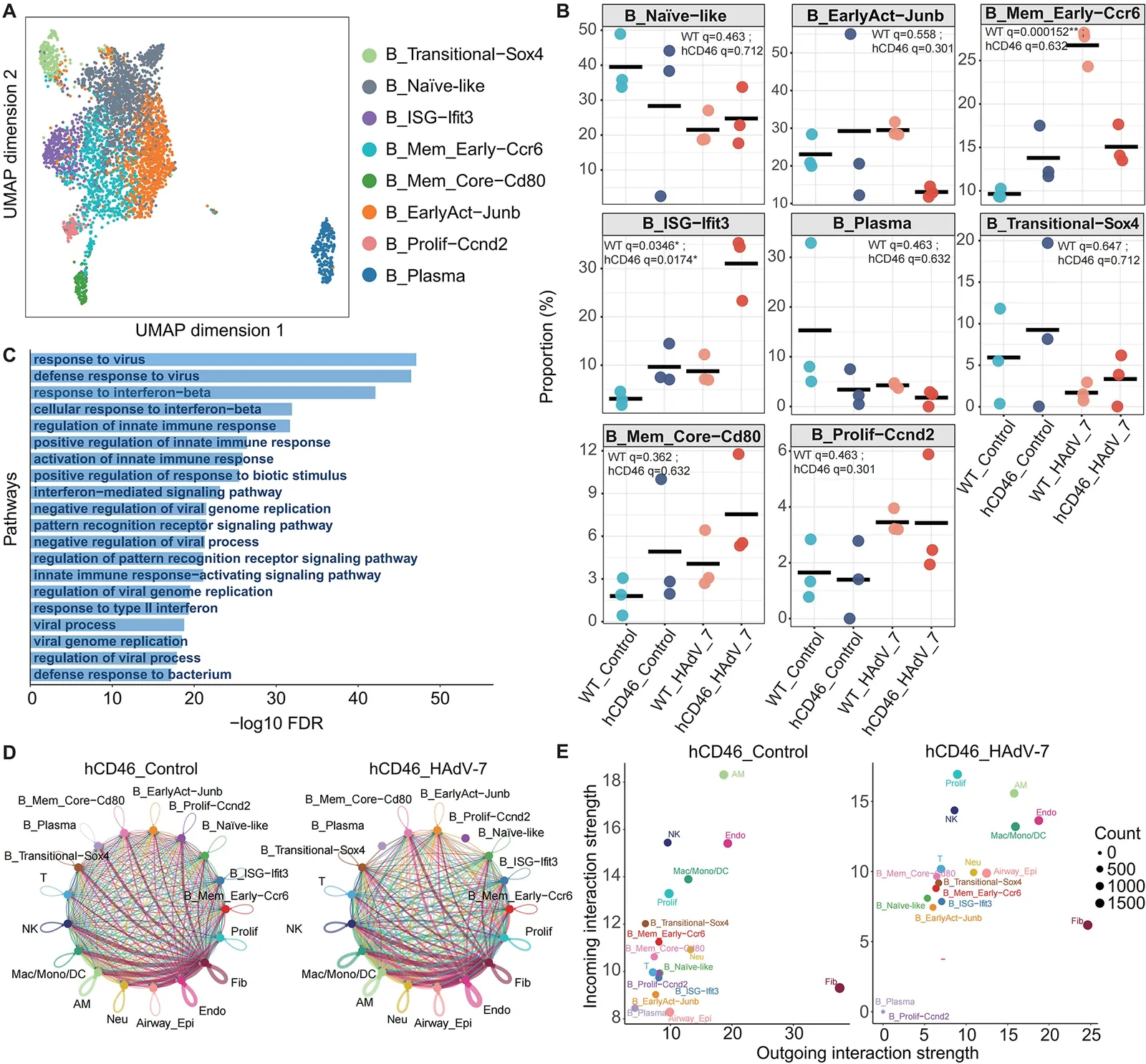
Interferon associated remodeling of pulmonary B cell states following HAdV-7 infection. **(A)** UMAP visualization of annotated B cell subpopulations across experimental groups; B cells comprised transitional B cells (B_Transitional–Sox4), Naïve-like B cells (B_Naïve-like), interferon stimulated B cells (B_ISG–Ifit3), early memory-like B cells (B_Mem_Early–Ccr6), core memory-like B cells (B_Mem_Core–Cd80), early activated B cells (B_EarlyAct–Junb), proliferating B cells (B_Prolif–Ccnd2), and plasma cells (B_Plasma). **(B)** Sample level proportions of B cell states across genotype and infection conditions. Each point represents one biologically independent mouse (n = 3 mice per group), and horizontal bars indicate group means. Cell proportions were analyzed using a linear model on logit transformed proportions with genotype, infection, and their interaction as predictors. q values represent Benjamini–Hochberg adjusted P values within the B cell compartment. **(C)** Top enriched pathways in B cells from hCD46_HAdV-7 versus hCD46 control lungs. **(D)** CellChat predicted ligand–receptor interaction networks involving B cell subsets and other pulmonary cell populations in hCD46_Control and hCD46_HAdV-7 lungs. Edge widths represent inferred interaction strength. **(E)** CellChat signaling role scatter plot showing incoming versus outgoing interaction strength of B cell subsets in hCD46_Control and hCD46_HAdV-7 lungs. *q < 0.05; **q < 0.01; ***q < 0.001.

### CellChat predicted intercellular signaling network remodeling in hCD46_HAdV-7 lungs

To further characterize changes in the pulmonary intercellular signaling landscape following HAdV-7 infection, we employed CellChat to infer ligand–receptor communication from the scRNA-seq dataset. Global analysis showed that hCD46_HAdV-7 lungs had the highest number of inferred interactions across all experimental groups (5, 319), whereas the aggregate interaction strength remained comparable to that of hCD46_Control lungs(100.01 versus 102.04; **Figure 6A**). Circle plots further showed extensive predicted connectivity among myeloid, lymphoid, and structural compartments in infected hCD46 lungs (**Figure 6B**; **Supplementary Figure S5A**). Signaling role analysis revealed marked changes in the network positions of several cell populations following infection. In hCD46_HAdV-7 lungs, Mono_Vcan and macrophage populations including Macro_Cxcl10, Macro_C1qa, and Macro_Gpnmb showed relatively high incoming and/or outgoing signaling scores. In particular, Macro_Cxcl10 and Macro_Gpnmb showed markedly higher signaling scores than in hCD46_Control lungs. Several structural populations, including endothelial cells and fibroblasts, also showed substantial inferred signaling activity. In contrast, T and B cells remained in the relatively low-centrality region of the inferred communication network, with both incoming and outgoing signaling strengths lower than those of the major myeloid and structural populations (**Figure 6C**). Differential interaction analysis further showed heterogeneous increases and decreases across sender–receiver pairs, indicating broad redistribution of predicted intercellular connectivity following HAdV-7 infection in hCD46_HAdV-7 lungs (**Figure 6D**). The accompanying marginal summaries showed that these changes involved multiple sender and receiver populations, including myeloid and structural compartments, rather than being restricted to a single cell type. Similar, although less pronounced, remodeling of the predicted communication network and increases in Macro_Cxcl10/Macro_Gpnmb signaling scores were also observed following infection in WT lungs (**Supplementary Figures S5B, C**). Overall, HAdV-7 infection was associated with broad remodeling of the predicted pulmonary communication network, with the most extensive interaction network observed in hCD46_HAdV-7 lungs and prominent changes involving selected inflammatory myeloid and structural populations. These network changes coincided with the interferon/ISG-associated myeloid and lymphoid transcriptional states described above, while T and B cells themselves remained relatively peripheral within the inferred communication network (**Figure 6E**).

**Figure 6:**
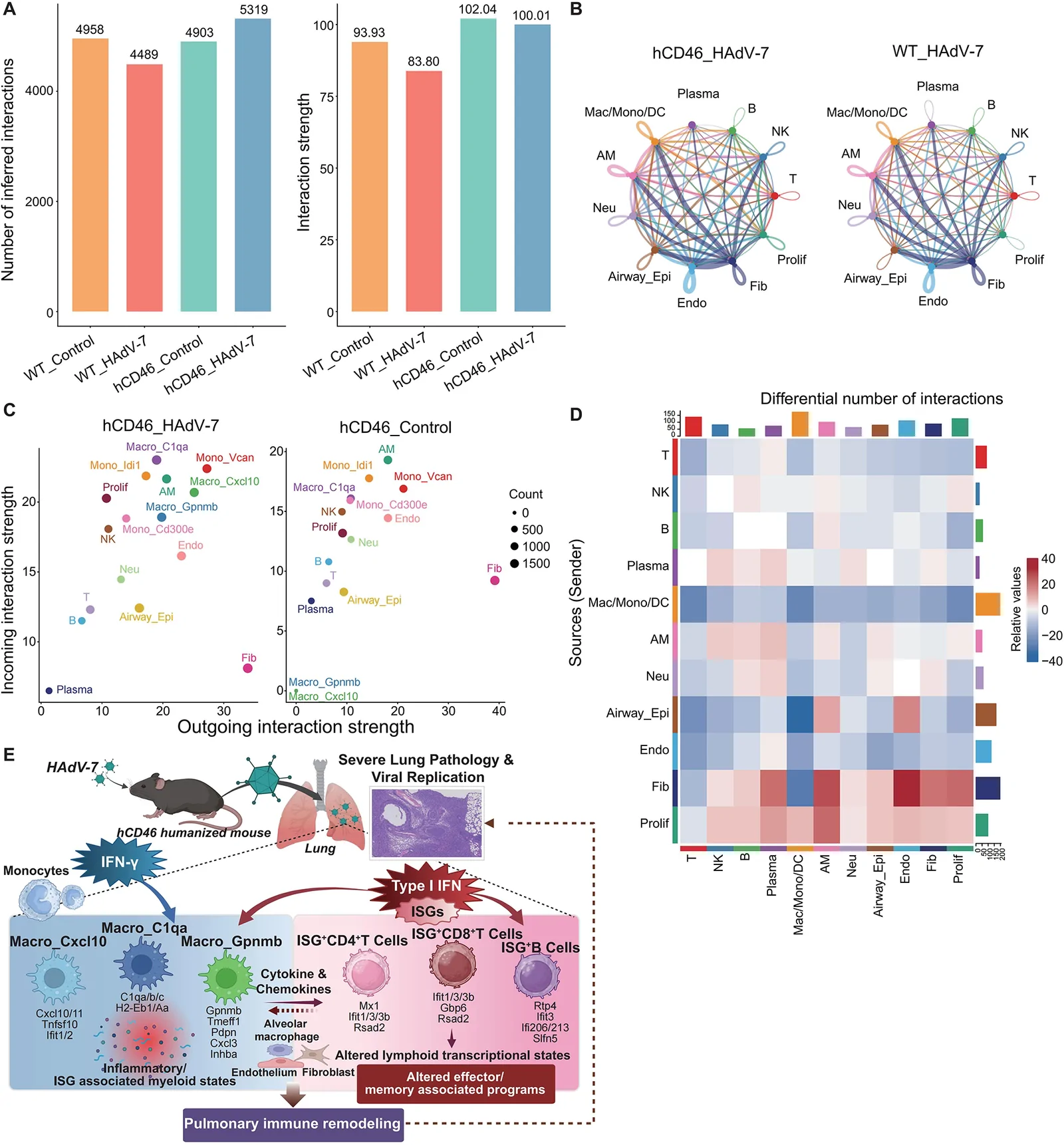
CellChat predicted intercellular signaling network remodeling in hCD46_HAdV-7 lungs. **(A)** Global comparison of interaction across groups. **(B)** CellChat predicted interaction networks among major pulmonary cell populations in hCD46_HAdV-7 and WT_HAdV-7 lungs. Edge widths represent inferred interaction strength. **(C)** CellChat derived outgoing and incoming signaling scores for pulmonary cell populations in hCD46_HAdV-7 and hCD46_Control lungs. Dot size indicates the number of cells represented in each population. **(D)** Heatmap of differential interactions comparing hCD46_HAdV-7 versus hCD46_Control lungs. Positive and negative values indicate relative increases and decreases in predicted interaction numbers, respectively. Top and right marginal bars summarize differential interaction counts associated with receiver and sender populations, respectively. **(E)** schematic summary integrating virological, transcriptional, and predicted intercellular communication features observed in hCD46_HAdV-7 lungs.

### Human PBMC transcriptomes show systemic concordance with selected immune programs identified in hCD46_HAdV-7 lungs

To assess whether selected immune programs identified in HAdV-7 infected hCD46 lungs were also detectable systemically during human HAdV infection, we analyzed PBMC bulk transcriptomes from pediatric patients with HAdV infection and healthy controls. Additional demographic and clinical characteristics, including age, sex, disease severity, ICU admission, treatments before PBMC sampling, length of hospital stay, and clinical outcomes, are summarized in **Supplementary Table S5**; among the 13 HAdV infected patients, 11 (84.6%) had severe disease, and all were discharged. Because viral typing information was unavailable in this cohort, this analysis was conservatively restricted to infection associated immune programs rather than HAdV-7 specific mechanisms. Clinical laboratory profiling revealed systemic abnormalities in infected patients, including reduced erythrocyte and hemoglobin related indices, decreased total protein and albumin levels, and increased LDH and AST levels, together with trends toward neutrophil skewed and lymphocyte reduced profiles (**Figure 7A**). Consistent with these clinical features, principal component analysis showed a clear transcriptomic separation between HAdV infected patients and healthy controls, indicating infection associated remodeling of the peripheral immune transcriptome (**Figure 7B**). Differential expression analysis further identified significant upregulation of several interferon stimulated and myeloid inflammation related genes in HAdV infected patients (**Supplementary Figure S6A**).

**Figure 7:**
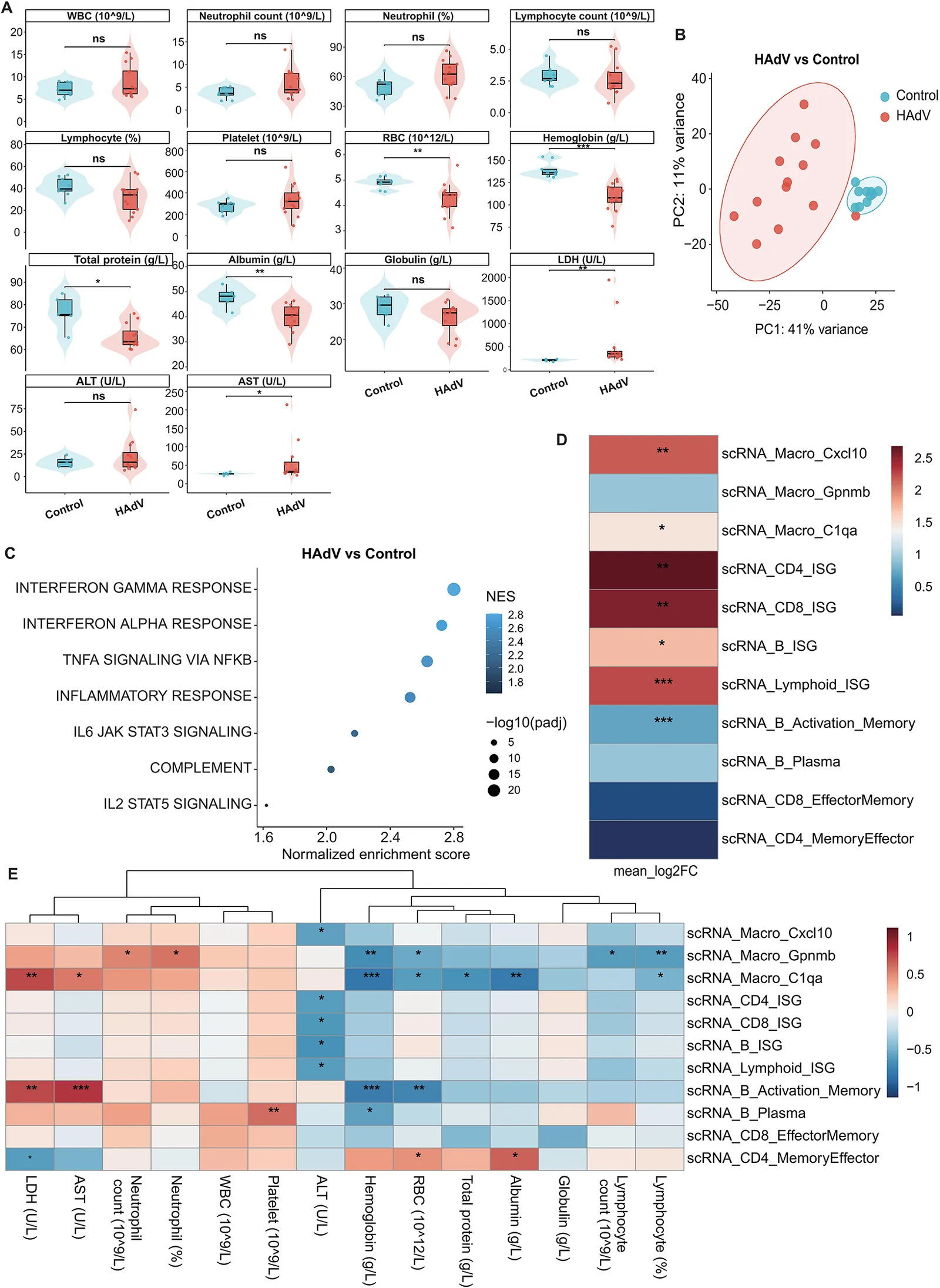
Human PBMC transcriptomes show systemic immune features concordant with selected mouse lung scRNA-seq derived immune programs. **(A)** Clinical laboratory indicators in pediatric patients with HAdV infection and healthy controls. Each point represents an individual sample. **(B)** Principal component analysis of PBMC bulk RNA-seq profiles from HAdV infected patients and healthy controls. **(C)** Hallmark gene set enrichment analysis of HAdV infected patients versus healthy controls using DESeq2 Wald statistics as the preranked metric. Dot size indicates −log10 adjusted P value, and color indicates normalized enrichment score. **(D)** Signature level concordance analysis showing the mean log_2_ fold change of genes from mouse lung scRNA-seq derived signatures in human HAdV versus control PBMC transcriptomes. Asterisks indicate significance assessed by one-sample Wilcoxon tests followed by Benjamini−Hochberg correction. **(E)** Spearman correlation heatmap showing exploratory associations between scRNA derived signature scores and commonly available laboratory indicators across all samples. Color indicates Spearman correlation coefficient. Asterisks indicate nominal Spearman P values.

Pathway level analysis further demonstrated broad activation of interferon and inflammation related programs in HAdV infected PBMCs, including interferon responses, TNF/NF-κB signaling, inflammatory response, IL6-JAK-STAT3 signaling, and complement associated pathways (**Figure 7C**). We next projected selected mouse lung scRNA-seq derived immune signatures onto the human PBMC transcriptomes. Signature level concordance analysis showed that genes comprising Macro_Cxcl10-like, Macro_Gpnmb-like, and Macro_C1qa-like mouse lung-derived myeloid signatures, and lymphoid ISG associated programs were directionally upregulated in HAdV infected patients compared with healthy controls (**Figure 7D**). Consistently, sample level signature scoring and core gene heatmaps further supported the activation of these scRNA derived inflammatory and ISG associated programs in HAdV infected PBMCs (**Supplementary Figures S6B, C**).

Exploratory clinical correlation analysis further linked these scRNA derived immune signatures with commonly available laboratory indicators. Across all samples, myeloid associated signatures were positively associated with neutrophil related and tissue injury related indices and inversely associated with erythrocyte/hemoglobin related, protein/albumin related, and lymphocyte related measures. In contrast, lymphoid memory/effector associated signatures tended to display an opposite pattern (**Figure 7E**). Together, these human PBMC data indicate that systemic interferon associated and myeloid inflammatory transcriptional features were concordant with selected immune programs identified in HAdV-7 infected hCD46 lungs.

## Discussion

HAdV-7 is a major etiological agent of severe pediatric pneumonia and represents a significant threat to child health worldwide (34). Despite its clinical importance, the mechanisms linking HAdV-7 infection to immune dysregulation and lung pathology remain poorly understood, largely due to the lack of physiologically relevant animal models (22, 35). HAdV-7 infects host cells through engagement of the CD46 receptor, which is absent from the murine respiratory tract (36). In the present study, hCD46 humanized mice showed higher pulmonary viral burden and more severe lung pathology following HAdV-7 infection. Lung homogenates from infected hCD46 mice also induced more pronounced cytopathic effects in A549 cells than those from infected WT mice, consistent with greater recoverable viral infectivity. Together, these findings support the utility of this model for studying HAdV-7 associated lung disease.

Using single-cell transcriptomic profiling, we further examined pulmonary immune changes associated with HAdV-7 infection in hCD46 humanized mice. Compared with WT_HAdV-7 mice, hCD46_HAdV-7 lungs showed type I and type II interferon associated transcriptional programs together with marked myeloid remodeling. Higher pulmonary viral burden was accompanied by reduced alveolar macrophages and increased inflammatory macrophage populations such as Macro_Cxcl10 and Macro_Gpnmb, which were enriched for interferon signaling, chemokine production, hypoxia adaptation, and lipid metabolism, resembling pathogenic macrophage states described in severe viral pneumonia (37, 38). Macro_Cxcl10 macrophages expressed canonical IFN-I stimulated genes together with IFN-γ associated chemokines such as Cxcl10 and Cxcl11, suggesting the coexistence of antiviral ISG programs and inflammatory chemokine signaling. Increasing evidence suggests that although early interferon signaling is protective, sustained or excessive activation may promote immunopathology rather than viral clearance in respiratory infections (39, 40). The prominent interferon associated programs observed here are consistent with this concept, although their direct contribution to lung injury remains to be experimentally established. These experimental observations are also consistent with our previous retrospective clinical observations in children with severe HAdV-7 community acquired pneumonia (33), in whom elevated serum viral loads were associated with disease severity and increased LDH and transaminase levels. Fatal cases also showed elevated circulating IFN-γ, CXCL10, and VEGF, with CXCL10 showing a strong positive correlation with viral load. The enrichment of Cxcl10 associated programs in pulmonary macrophages observed here provides a potential cellular correlate of these systemic inflammatory findings, but does not establish the cellular source of circulating CXCL10. Likewise, elevated IFN-γ levels in the clinical cohort are consistent with our finding that IFN-II responses co-occurred with broader IFN-I associated ISG programs.The increase in circulating VEGF is also consistent with potential vascular involvement suggested by our communication network analysis. In hCD46_HAdV-7 lungs, endothelial cells showed substantial inferred signaling activity within the CellChat network, suggesting involvement of vascular and barrier related compartments in pulmonary immune remodeling. Supporting this interpretation, our previous *in vitro* studies demonstrated that HAdV-7 can directly infect pulmonary endothelial cells and undergo productive replication, accompanied by significant induction of VEGF expression (41). These observations provide additional context for a potential association between vascular activation and systemic inflammation. In addition, CellChat analysis predicted prominent signaling roles for selected inflammatory macrophage and stromal populations, with broad remodeling of the inferred intercellular communication network. Fibroblasts also showed relatively high inferred outgoing signaling activity, consistent with prior studies describing lung fibroblasts as stromal sentinels that coordinate inflammatory signaling through cytokine and chemokine secretion (42–44).

Analysis of lymphocyte populations showed coordinated changes across B cells, CD4^^+^^ T cells, and CD8^^+^^ T cells. In hCD46 mice, the CD4^^+^^ T_ISG–Mx1 population showed an increasing trend following infection, whereas CD4^^+^^ T_Treg–Il1rl1 increased significantly; naïve and central memory CD4^^+^^ T cell proportions showed no significant infection associated changes. CD8^^+^^ T_ISG–Ifit1 increased significantly following infection in both WT and hCD46 mice, with a greater magnitude of increase in hCD46_HAdV-7 mice than in WT_HAdV-7 mice, whereas the other CD8^^+^^ T cell states showed no significant infection associated changes. B cells also showed prominent ISG associated transcriptional programs, with B_ISG–Ifit3 increasing following infection in both genotypes, whereas B_Mem_Early–Ccr6 increased markedly in WT mice but showed no comparable increase in hCD46 mice. Collectively, these findings suggest that HAdV-7 infection was associated with altered lymphoid transcriptional states and prominent interferon/ISG programs. Although the human PBMC cohort lacked viral serotyping and could not directly validate lung resident mechanisms, concordant interferon associated myeloid and lymphoid ISG signatures were also detectable systemically in pediatric HAdV infection, providing clinical context for selected immune programs observed in the mouse lung.

Previous receptor humanized HAdV studies have mainly focused on receptor usage, viral burden, tissue pathology, endothelial injury, or therapeutic efficacy (21, 41). Our study extends these observations by characterizing pulmonary immune cell changes following HAdV-7 infection at single-cell resolution, providing useful reference endpoints for future immune and intervention studies using this model. Several limitations should be considered. First, hCD46 represents only one component of HAdV-7 receptor usage and does not fully reproduce the contribution of other human receptors, particularly the synergistic role of CD46 and DSG2 (45). The present study also examined a limited viral dose and a single major sampling time point, restricting assessment of dose-dependent and temporal immune changes. In addition, the pediatric PBMC cohort lacked viral serotyping, and peripheral blood transcriptomes cannot directly resolve lung specific cellular responses. Second, although selected lymphoid phenotypes and macrophage transcripts were independently assessed by flow cytometry and RT-qPCR, respectively, the major scRNA-seq derived immune states and signaling programs were not comprehensively validated at the protein or cytokine/chemokine level. Spatial transcriptomics or related tissue based approaches will also be required to validate the localization and cellular interactions predicted by the transcriptomic analyses. Finally, functional perturbation experiments were not performed; therefore, the present data cannot establish whether the identified interferon associated, myeloid, and lymphoid changes directly contribute to disease severity. Future studies incorporating these approaches, together with different viral doses, longitudinal sampling, additional receptor humanized models, and virus-typed clinical cohorts, will help further validate these observations.

## Conclusions

In conclusion, hCD46 humanized mice showed increased pulmonary viral burden and more severe lung pathology following HAdV-7 infection. Single-cell analysis revealed prominent interferon associated myeloid remodeling, predicted intercellular communication network changes involving myeloid and structural populations, and lymphoid ISG associated transcriptional states, with selected corresponding immune programs also detectable systemically in pediatric HAdV infection. These findings support the utility of this model for studying HAdV-7 associated lung disease and for future immune and intervention studies.

## Data Availability

The datasets presented in this study are available in the National Genomics Data Center (NGDC), China National Center for Bioinformation / Beijing Institute of Genomics, Chinese Academy of Sciences. The mouse lung scRNA-seq data have been deposited in the Genome Sequence Archive (GSA) under accession number CRA041299 (BioProject PRJCA061804) and are publicly searchable and downloadable at https://ngdc.cncb.ac.cn/gsa/browse/CRA041299. The human PBMC bulk RNA-seq data have been deposited in the Genome Sequence Archive for Human (GSA-Human) under accession number HRA021303 (BioProject PRJCA073862). The accession record is publicly searchable at https://ngdc.cncb.ac.cn/gsa-human/browse/HRA021303. The human dataset is available under controlled access, and researchers may request access through the GSA-Human portal.
