# Supplementary material for: Human CD46 humanized mice reveal interferon associated pulmonary immune remodeling during human adenovirus type 7 lung infection
Source: Front Immunol. 2026 Oct 13;17:1919716. doi: 10.3389/fimmu.2026.1919716 (PMC13710330; doi:10.3389/fimmu.2026.1919716)
Supplement: Supplementary file 1 [file DataSheet1.docx]

Supplementary Material

**Supplementary Figure S1.** (A) Distribution of total RNA counts per cell (nCount_RNA) across individual samples. (B) Distribution of detected genes per cell (nFeature_RNA) across individual samples. (C) Percentage of mitochondrial gene expression (percent.mt) across individual samples. (D) Dot plot showing canonical marker-gene expression across major pulmonary cell populations. (E) Gene expression scatter plot comparing WT_HAdV-7 and WT_Control lungs, highlighting representative differentially expressed genes. (F) Top enriched biological pathways in WT_HAdV-7 versus WT_Control lungs.

**Supplementary Figure S2.** (A) Dot plot of marker gene expression across monocyte and macrophage clusters. (B) Top 20 enriched pathways in myeloid populations from WT lungs (WT_HAdV-7 versus WT_Control). (C) RT-qPCR validation of interferon- and chemokine-associated genes in magnetically enriched pulmonary macrophages from hCD46 control and HAdV-7-infected mice at 5 dpi. HAdV-7 infection significantly increased expression of Cxcl10 (P = 0.0127), Cxcl11 (P = 0.0158), Ifit1 (P = 0.0028), Irf7 (P = 0.0024), Isg15 (P = 0.0474), and Stat1 (P = 0.0043). Statistical comparisons were performed using two-tailed unpaired t tests with Welch’s correction. *, P < 0.05; **, P < 0.01. (D) CellChat predicted interaction networks of interaction strength in WT lungs. (E) CellChat signaling role scatter plot (incoming vs outgoing interaction strength) in WT lungs.

**Supplementary Figure S3.** (A) Dot plot showing representative marker gene expression across annotated CD4^⁺^ T cell subpopulations. Dot size indicates the percentage of cells expressing each gene, and color intensity indicates average expression. (B) Top 20 enriched pathways in CD4^⁺^ T cells from hCD46_HAdV-7 versus hCD46_control lungs. (C) Top 20 enriched pathways in CD4^⁺^ T cells from WT_HAdV-7 versus WT_control lungs. (D) Dot plot showing representative marker gene expression across annotated CD8^⁺^ T cell subpopulations. (E) Top 20 enriched pathways in CD8^⁺^ T cells from hCD46_HAdV-7 versus hCD46_Control lungs. (F) Top 20 enriched pathways in CD8^⁺^ T cells from WT_HAdV-7 versus WT_Control lungs. (G) Flow cytometric validation of pulmonary T cell populations at 5 dpi. Representative plots show CD3^⁺^ T cell gating in WT_Control, WT_HAdV-7, hCD46_Control, and hCD46_HAdV-7 mice. Numbers indicate the percentage of CD3^⁺^ T cells among CD45^⁺^ cells. Quantification of total CD3^⁺^ T cells, CD4^⁺^ T cells, CD8^⁺^ T cells, CD4^⁺^ central memory-like (Tcm-like) cells, and CD8^⁺^ effector memory-like (Tem-like) cells is shown. WT_Control, n = 4; WT_HAdV-7, n = 5; hCD46_Control, n = 5; hCD46_HAdV-7, n = 5 biologically independent mice. Data are presented as mean ± SEM. Statistical comparisons were performed as described in the Methods. *, P < 0.05; **, P < 0.01; ** *, P < 0.001; ns, not significant.

**Supplementary Figure S4.** (A) Dot plot showing marker gene expression across annotated B cell subpopulations, supporting cell type annotation. (B) Flowcytometric assessment of pulmonary B cell populations at 5 dpi, including total CD19⁺ B cells, CD19⁺CD138⁺ plasma-like cells, and MHC-II⁺ B cells. WT_Control, n = 4; WT_HAdV-7, n = 5; hCD46_Control, n = 5; and hCD46_HAdV-7, n = 5 biologically independent mice. Data are presented as mean ± SEM. Statistical comparisons were performed as described in the Methods. ns, not significant. (C) Top 20 enriched pathways in B cells from WT_HAdV-7 versus WT_control lungs. (D) Monocle3 inferred B cell trajectory colored by pseudotime. B_ISG–Ifit3 cells were positioned predominantly within the mid-to-late region of the inferred trajectory in infected hCD46 lungs. (E) CellChat predicted ligand–receptor interaction networks involving B cell subsets and other pulmonary populations in WT_Control and WT_HAdV-7 lungs. Edge widths represent inferred interaction strength.

**Supplementary Figure S5.** (A) CellChat predicted interaction networks among major pulmonary cell populations in hCD46_Control and WT_Control lungs. Edge widths represent inferred interaction strength. (B) CellChat derived outgoing and incoming signaling scores for pulmonary cell populations in WT_HAdV-7 and WT_Control lungs. Dot size indicates the number of cells represented in each population. (C) Heatmap showing relative differences in the number of predicted interactions between WT_HAdV-7 and WT_Control lungs across sender–receiver pairs. Positive and negative values indicate relative increases and decreases in predicted interaction numbers, respectively. Top and right marginal bars summarize differential interaction counts associated with receiver and sender populations, respectively.

**Supplementary Figure S6.** (A) Volcano plot showing differentially expressed genes between HAdV infected patients and healthy controls in PBMC bulk RNA-seq data. Genes with adjusted P value <0.05 and absolute log2 fold change >1 were considered significantly differentially expressed. Selected interferon stimulated, myeloid inflammatory, and lymphoid-associated genes are labeled. (B) Sample level scores of mouse lung scRNA-seq derived signatures projected onto human PBMC transcriptomes. Signature scores were calculated as the mean z-score of detected signature genes. Statistical significance was assessed using two-sided Wilcoxon rank sum tests followed by Benjamini Hochberg correction. (C) Heatmap showing the expression of representative core genes from each mouse lung scRNA-seq derived signature in human PBMC samples. Up to five detected genes per signature are displayed. Expression values were z-score scaled across samples for each gene. Columns represent individual samples ordered by group, and rows represent signature genes grouped by their corresponding scRNA derived modules.

**Supplementary Table S1.** Primers used in this study.

| **Gene** | **Forward (5' → 3')** | **Reverse (5' → 3')** |
| --- | --- | --- |
| Hexon | GCCCCARTGGGCRTACATGCACATC | AGCACSCCSCGRATGTCAAAG |
| Cxcl10 | ATCATCCCTGCGAGCCTATCCT | GACCTTTTTTGGCTAAACGCTTTC |
| Cxcl11 | CCGAGTAACGGCTGCGACAAAG | CCTGCATTATGAGGCGAGCTTG |
| Isg15 | CATCCTGGTGAGGAACGAAAGG | CTCAGCCAGAACTGGTCTTCGT |
| Ifit1 | TACAGGCTGGAGTGTGCTGAGA | CTCCACTTTCAGAGCCTTCGCA |
| Irf7 | CCTCTGCTTTCTAGTGATGCCG | CGTAAACACGGTCTTGCTCCTG |
| Stat1 | GCCTCTCATTGTCACCGAAGAAC | TGGCTGACGTTGGAGATCACCA |
| Gapdh | TGGCATTGTTACCAACTGGGAC | TCACGGTTGGCCTTAGGGTTC |

**Supplementary Table S2.** Top 20 marker genes for CD4⁺ T cell subclusters identified by lung scRNA-seq analysis.

|  | **p_value** | **avg_log2FC** | **pct.1** | **pct.2** | **p_value_adj** | **cluster** | **gene** |
| --- | --- | --- | --- | --- | --- | --- | --- |
| 1 | 0.000E+00 | 2.726 | 0.775 | 0.149 | 0.000E+00 | CD4_Naïve-Igfbp4 | Ccr7 |
| 2 | 0.000E+00 | 3.117 | 0.768 | 0.148 | 0.000E+00 | CD4_Naïve-Igfbp4 | Ifngr2 |
| 3 | 0.000E+00 | 5.475 | 0.638 | 0.025 | 0.000E+00 | CD4_Naïve-Igfbp4 | Igfbp4 |
| 4 | 0.000E+00 | 2.749 | 0.752 | 0.164 | 0.000E+00 | CD4_Naïve-Igfbp4 | Actn1 |
| 5 | 0.000E+00 | 2.875 | 0.722 | 0.135 | 0.000E+00 | CD4_Naïve-Igfbp4 | Patj |
| 6 | 0.000E+00 | 3.181 | 0.645 | 0.076 | 0.000E+00 | CD4_Naïve-Igfbp4 | St8sia6 |
| 7 | 0.000E+00 | 2.894 | 0.993 | 0.454 | 0.000E+00 | CD4_Naïve-Igfbp4 | Lef1 |
| 8 | 0.000E+00 | 3.086 | 0.672 | 0.145 | 0.000E+00 | CD4_Naïve-Igfbp4 | Slc49a4 |
| 9 | 0.000E+00 | 4.256 | 0.535 | 0.036 | 0.000E+00 | CD4_Naïve-Igfbp4 | Atp1b1 |
| 10 | 0.000E+00 | 4.541 | 0.384 | 0.018 | 0.000E+00 | CD4_Naïve-Igfbp4 | Dapl1 |
| 11 | 3.673E-294 | 2.627 | 0.763 | 0.229 | 9.256E-290 | CD4_Naïve-Igfbp4 | Rras2 |
| 12 | 1.365E-254 | 2.840 | 0.688 | 0.214 | 3.440E-250 | CD4_Naïve-Igfbp4 | Ndrg3 |
| 13 | 1.312E-170 | 2.763 | 0.402 | 0.080 | 3.306E-166 | CD4_Naïve-Igfbp4 | Cers6 |
| 14 | 1.628E-157 | 2.816 | 0.319 | 0.049 | 4.103E-153 | CD4_Naïve-Igfbp4 | Myo10 |
| 15 | 6.625E-155 | 3.027 | 0.313 | 0.048 | 1.670E-150 | CD4_Naïve-Igfbp4 | Maml3 |
| 16 | 2.945E-140 | 2.963 | 0.290 | 0.045 | 7.421E-136 | CD4_Naïve-Igfbp4 | 1810041H14Rik |
| 17 | 3.499E-140 | 2.804 | 0.285 | 0.042 | 8.816E-136 | CD4_Naïve-Igfbp4 | Vipr1 |
| 18 | 1.487E-138 | 2.768 | 0.298 | 0.049 | 3.746E-134 | CD4_Naïve-Igfbp4 | Treml2 |
| 19 | 6.157E-133 | 2.869 | 0.266 | 0.039 | 1.551E-128 | CD4_Naïve-Igfbp4 | Gm52870 |
| 20 | 2.198E-123 | 2.732 | 0.303 | 0.060 | 5.538E-119 | CD4_Naïve-Igfbp4 | Itm2a |
| 21 | 2.939E-155 | 1.844 | 0.819 | 0.371 | 7.407E-151 | CD4_Tcm-Slamf6 | Slamf6 |
| 22 | 8.163E-134 | 1.670 | 0.844 | 0.422 | 2.057E-129 | CD4_Tcm-Slamf6 | Adk |
| 23 | 9.298E-132 | 1.467 | 0.909 | 0.471 | 2.343E-127 | CD4_Tcm-Slamf6 | Aff3 |
| 24 | 1.017E-131 | 1.991 | 0.714 | 0.301 | 2.563E-127 | CD4_Tcm-Slamf6 | Tbc1d4 |
| 25 | 1.313E-119 | 1.707 | 0.845 | 0.494 | 3.308E-115 | CD4_Tcm-Slamf6 | Asap1 |
| 26 | 1.560E-65 | 1.216 | 0.705 | 0.384 | 3.931E-61 | CD4_Tcm-Slamf6 | St6gal1 |
| 27 | 3.284E-64 | 1.839 | 0.479 | 0.206 | 8.274E-60 | CD4_Tcm-Slamf6 | Pgap1 |
| 28 | 7.853E-62 | 1.354 | 0.615 | 0.320 | 1.979E-57 | CD4_Tcm-Slamf6 | Casp4 |
| 29 | 1.503E-61 | 1.476 | 0.650 | 0.362 | 3.788E-57 | CD4_Tcm-Slamf6 | Eea1 |
| 30 | 3.280E-60 | 1.308 | 0.472 | 0.188 | 8.267E-56 | CD4_Tcm-Slamf6 | Rflnb |
| 31 | 2.218E-58 | 1.682 | 0.373 | 0.134 | 5.589E-54 | CD4_Tcm-Slamf6 | Tnfrsf26 |
| 32 | 2.645E-54 | 1.551 | 0.507 | 0.248 | 6.665E-50 | CD4_Tcm-Slamf6 | Shld1 |
| 33 | 1.424E-51 | 1.870 | 0.295 | 0.097 | 3.589E-47 | CD4_Tcm-Slamf6 | Myb |
| 34 | 2.410E-51 | 1.294 | 0.413 | 0.166 | 6.072E-47 | CD4_Tcm-Slamf6 | Nsg2 |
| 35 | 1.020E-49 | 1.310 | 0.432 | 0.185 | 2.572E-45 | CD4_Tcm-Slamf6 | Izumo1r |
| 36 | 1.987E-46 | 1.700 | 0.332 | 0.126 | 5.008E-42 | CD4_Tcm-Slamf6 | Slc9a7 |
| 37 | 8.533E-45 | 1.520 | 0.541 | 0.304 | 2.150E-40 | CD4_Tcm-Slamf6 | Igf1r |
| 38 | 6.834E-44 | 1.211 | 0.558 | 0.312 | 1.722E-39 | CD4_Tcm-Slamf6 | Btla |
| 39 | 3.280E-41 | 1.582 | 0.267 | 0.093 | 8.265E-37 | CD4_Tcm-Slamf6 | B630019A10Rik |
| 40 | 2.755E-25 | 1.136 | 0.332 | 0.168 | 6.942E-21 | CD4_Tcm-Slamf6 | Gm36279 |
| 41 | 8.352E-12 | 0.894 | 0.882 | 0.851 | 2.105E-07 | CD4_Resting-Sh3bgrl3 | Sh3bgrl3 |
| 42 | 1.062E-09 | 0.897 | 0.791 | 0.745 | 2.677E-05 | CD4_Resting-Sh3bgrl3 | Dynll1 |
| 43 | 3.020E-08 | 0.925 | 0.736 | 0.615 | 7.611E-04 | CD4_Resting-Sh3bgrl3 | Atp6v1f |
| 44 | 8.581E-08 | 1.265 | 0.600 | 0.459 | 2.162E-03 | CD4_Resting-Sh3bgrl3 | Mrps21 |
| 45 | 2.169E-07 | 1.202 | 0.645 | 0.545 | 5.466E-03 | CD4_Resting-Sh3bgrl3 | Tomm6 |
| 46 | 1.684E-06 | 0.956 | 0.655 | 0.621 | 4.244E-02 | CD4_Resting-Sh3bgrl3 | Cox5b |
| 47 | 9.551E-06 | 0.888 | 0.709 | 0.713 | 2.407E-01 | CD4_Resting-Sh3bgrl3 | Atp5g2 |
| 48 | 9.578E-06 | 0.980 | 0.682 | 0.627 | 2.414E-01 | CD4_Resting-Sh3bgrl3 | Cox5a |
| 49 | 2.547E-05 | 0.997 | 0.609 | 0.551 | 6.419E-01 | CD4_Resting-Sh3bgrl3 | Ndufa3 |
| 50 | 3.355E-05 | 1.074 | 0.573 | 0.459 | 8.454E-01 | CD4_Resting-Sh3bgrl3 | Lamtor2 |
| 51 | 4.197E-05 | 0.969 | 0.573 | 0.483 | 1.000E+00 | CD4_Resting-Sh3bgrl3 | Timm13 |
| 52 | 4.630E-05 | 1.028 | 0.636 | 0.576 | 1.000E+00 | CD4_Resting-Sh3bgrl3 | Aldoa |
| 53 | 8.699E-05 | 0.908 | 0.673 | 0.662 | 1.000E+00 | CD4_Resting-Sh3bgrl3 | Sec61g |
| 54 | 1.480E-04 | 0.984 | 0.545 | 0.515 | 1.000E+00 | CD4_Resting-Sh3bgrl3 | Atp5k |
| 55 | 6.127E-04 | 0.920 | 0.573 | 0.574 | 1.000E+00 | CD4_Resting-Sh3bgrl3 | Ran |
| 56 | 2.728E-03 | 1.274 | 0.300 | 0.208 | 1.000E+00 | CD4_Resting-Sh3bgrl3 | Trappc2l |
| 57 | 3.670E-03 | 1.094 | 0.427 | 0.373 | 1.000E+00 | CD4_Resting-Sh3bgrl3 | Ndufc2 |
| 58 | 6.595E-03 | 1.179 | 0.309 | 0.232 | 1.000E+00 | CD4_Resting-Sh3bgrl3 | Psmg4 |
| 59 | 7.133E-03 | 0.889 | 0.564 | 0.570 | 1.000E+00 | CD4_Resting-Sh3bgrl3 | Mif |
| 60 | 9.839E-03 | 1.137 | 0.300 | 0.230 | 1.000E+00 | CD4_Resting-Sh3bgrl3 | Guk1 |
| 61 | 6.411E-189 | 2.037 | 0.565 | 0.173 | 1.616E-184 | CD4_Th1-Ifng | Nkg7 |
| 62 | 1.566E-184 | 2.343 | 0.516 | 0.140 | 3.946E-180 | CD4_Th1-Ifng | Tbx21 |
| 63 | 1.090E-181 | 1.999 | 0.478 | 0.117 | 2.746E-177 | CD4_Th1-Ifng | Ifng |
| 64 | 5.848E-169 | 3.319 | 0.331 | 0.052 | 1.474E-164 | CD4_Th1-Ifng | Itga1 |
| 65 | 2.371E-163 | 3.317 | 0.629 | 0.308 | 5.976E-159 | CD4_Th1-Ifng | Ccl5 |
| 66 | 1.212E-157 | 2.174 | 0.493 | 0.152 | 3.055E-153 | CD4_Th1-Ifng | Ctsw |
| 67 | 1.564E-136 | 1.392 | 0.765 | 0.466 | 3.940E-132 | CD4_Th1-Ifng | Sorl1 |
| 68 | 9.757E-112 | 2.530 | 0.309 | 0.074 | 2.459E-107 | CD4_Th1-Ifng | Dock5 |
| 69 | 6.612E-110 | 1.703 | 0.455 | 0.162 | 1.666E-105 | CD4_Th1-Ifng | Cxcr3 |
| 70 | 4.446E-93 | 2.245 | 0.366 | 0.127 | 1.120E-88 | CD4_Th1-Ifng | Il12rb2 |
| 71 | 5.283E-82 | 1.511 | 0.530 | 0.274 | 1.331E-77 | CD4_Th1-Ifng | Abcb1a |
| 72 | 3.217E-74 | 1.515 | 0.463 | 0.218 | 8.106E-70 | CD4_Th1-Ifng | Hip1 |
| 73 | 3.993E-69 | 1.676 | 0.357 | 0.142 | 1.006E-64 | CD4_Th1-Ifng | Chst11 |
| 74 | 6.219E-68 | 1.530 | 0.472 | 0.246 | 1.567E-63 | CD4_Th1-Ifng | Il18rap |
| 75 | 4.104E-62 | 1.721 | 0.341 | 0.145 | 1.034E-57 | CD4_Th1-Ifng | Fasl |
| 76 | 2.824E-58 | 1.947 | 0.270 | 0.098 | 7.116E-54 | CD4_Th1-Ifng | Nmnat2 |
| 77 | 8.900E-58 | 1.509 | 0.411 | 0.208 | 2.243E-53 | CD4_Th1-Ifng | Sntb2 |
| 78 | 9.092E-45 | 1.437 | 0.379 | 0.209 | 2.291E-40 | CD4_Th1-Ifng | P2rx7 |
| 79 | 2.419E-37 | 1.366 | 0.492 | 0.346 | 6.096E-33 | CD4_Th1-Ifng | Lyst |
| 80 | 2.630E-31 | 1.454 | 0.371 | 0.228 | 6.628E-27 | CD4_Th1- | Abcb1b |
| 81 | 2.619E-60 | 2.474 | 0.270 | 0.046 | 6.599E-56 | CD4_Th2-Gata3 | Il1rl1 |
| 82 | 1.475E-58 | 1.741 | 0.546 | 0.178 | 3.716E-54 | CD4_Th2-Gata3 | Ccr4 |
| 83 | 5.423E-55 | 1.442 | 0.743 | 0.355 | 1.367E-50 | CD4_Th2-Gata3 | Ramp3 |
| 84 | 4.778E-53 | 2.547 | 0.279 | 0.056 | 1.204E-48 | CD4_Th2-Gata3 | Cysltr1 |
| 85 | 8.614E-48 | 1.465 | 0.759 | 0.401 | 2.171E-43 | CD4_Th2-Gata3 | Cd40lg |
| 86 | 2.870E-44 | 1.673 | 0.568 | 0.231 | 7.232E-40 | CD4_Th2-Gata3 | Ar |
| 87 | 1.935E-39 | 1.619 | 0.498 | 0.197 | 4.876E-35 | CD4_Th2-Gata3 | Nav2 |
| 88 | 7.036E-37 | 1.563 | 0.533 | 0.228 | 1.773E-32 | CD4_Th2-Gata3 | Acvr2a |
| 89 | 8.202E-37 | 1.667 | 0.648 | 0.350 | 2.067E-32 | CD4_Th2-Gata3 | Gata3 |
| 90 | 6.340E-36 | 2.264 | 0.419 | 0.164 | 1.598E-31 | CD4_Th2-Gata3 | Kcnq5 |
| 91 | 3.479E-34 | 1.936 | 0.311 | 0.094 | 8.768E-30 | CD4_Th2-Gata3 | Itga2 |
| 92 | 7.181E-31 | 1.614 | 0.467 | 0.207 | 1.810E-26 | CD4_Th2-Gata3 | Frmd4b |
| 93 | 1.275E-29 | 1.668 | 0.394 | 0.157 | 3.213E-25 | CD4_Th2-Gata3 | Chn2 |
| 94 | 1.458E-28 | 1.845 | 0.298 | 0.100 | 3.673E-24 | CD4_Th2-Gata3 | 4930532G15Rik |
| 95 | 1.181E-25 | 2.187 | 0.321 | 0.125 | 2.977E-21 | CD4_Th2-Gata3 | Atf3 |
| 96 | 9.158E-25 | 1.415 | 0.505 | 0.260 | 2.308E-20 | CD4_Th2-Gata3 | Glb1 |
| 97 | 5.479E-24 | 1.643 | 0.470 | 0.240 | 1.381E-19 | CD4_Th2-Gata3 | Lmo4 |
| 98 | 8.359E-24 | 1.739 | 0.330 | 0.130 | 2.106E-19 | CD4_Th2-Gata3 | Hk2 |
| 99 | 6.401E-23 | 1.559 | 0.270 | 0.096 | 1.613E-18 | CD4_Th2-Gata3 | Ccr6 |
| 100 | 1.125E-15 | 1.555 | 0.425 | 0.242 | 2.836E-11 | CD4_Th2-Gata3 | Ptpn13 |
| 101 | 0.000E+00 | 5.409 | 0.542 | 0.040 | 0.000E+00 | CD4_Th17-Il17a | Il17a |
| 102 | 0.000E+00 | 4.724 | 0.437 | 0.022 | 0.000E+00 | CD4_Th17-Il17a | Ppp1r14c |
| 103 | 2.917E-299 | 3.083 | 0.676 | 0.141 | 7.350E-295 | CD4_Th17-Il17a | Tmem176a |
| 104 | 3.674E-230 | 4.271 | 0.389 | 0.043 | 9.258E-226 | CD4_Th17-Il17a | Igfbp7 |
| 105 | 1.743E-222 | 3.941 | 0.340 | 0.029 | 4.392E-218 | CD4_Th17-Il17a | Ablim3 |
| 106 | 1.485E-219 | 4.113 | 0.339 | 0.030 | 3.741E-215 | CD4_Th17-Il17a | Cpe |
| 107 | 6.847E-203 | 3.147 | 0.457 | 0.081 | 1.725E-198 | CD4_Th17-Il17a | Ckb |
| 108 | 2.930E-201 | 3.181 | 0.368 | 0.044 | 7.385E-197 | CD4_Th17-Il17a | Rorc |
| 109 | 9.172E-201 | 3.758 | 0.354 | 0.040 | 2.311E-196 | CD4_Th17-Il17a | Il23r |
| 110 | 2.280E-198 | 3.039 | 0.420 | 0.065 | 5.744E-194 | CD4_Th17-Il17a | Il1r1 |
| 111 | 1.348E-195 | 3.604 | 0.347 | 0.040 | 3.396E-191 | CD4_Th17-Il17a | Havcr2 |
| 112 | 1.805E-174 | 2.855 | 0.443 | 0.088 | 4.549E-170 | CD4_Th17-Il17a | Adam12 |
| 113 | 7.227E-167 | 2.975 | 0.359 | 0.055 | 1.821E-162 | CD4_Th17-Il17a | Sdc4 |
| 114 | 1.905E-160 | 3.260 | 0.298 | 0.035 | 4.801E-156 | CD4_Th17-Il17a | 1700012B07Rik |
| 115 | 3.147E-145 | 3.486 | 0.367 | 0.073 | 7.930E-141 | CD4_Th17-Il17a | Il1r2 |
| 116 | 6.792E-144 | 2.811 | 0.308 | 0.045 | 1.712E-139 | CD4_Th17-Il17a | Gm36347 |
| 117 | 4.779E-137 | 3.315 | 0.255 | 0.030 | 1.204E-132 | CD4_Th17-Il17a | Gm49890 |
| 118 | 4.283E-134 | 3.030 | 0.255 | 0.030 | 1.079E-129 | CD4_Th17-Il17a | Il17re |
| 119 | 9.305E-134 | 3.123 | 0.269 | 0.036 | 2.345E-129 | CD4_Th17-Il17a | Ccr1 |
| 120 | 1.911E-120 | 3.141 | 0.295 | 0.053 | 4.817E-116 | CD4_Th17-Il17a | Lag3 |
| 121 | 1.614E-180 | 4.839 | 0.466 | 0.024 | 4.067E-176 | CD4_Treg_Act-Lrrc32 | Gm4956 |
| 122 | 4.485E-141 | 4.032 | 0.473 | 0.035 | 1.130E-136 | CD4_Treg_Act-Lrrc32 | Lrrc32 |
| 123 | 1.287E-105 | 2.561 | 0.635 | 0.084 | 3.244E-101 | CD4_Treg_Act-Lrrc32 | Foxp3 |
| 124 | 6.690E-105 | 3.393 | 0.838 | 0.182 | 1.686E-100 | CD4_Treg_Act-Lrrc32 | Ift80 |
| 125 | 2.489E-98 | 5.298 | 0.270 | 0.015 | 6.273E-94 | CD4_Treg_Act-Lrrc32 | Gpr83 |
| 126 | 1.176E-57 | 2.163 | 0.858 | 0.320 | 2.963E-53 | CD4_Treg_Act-Lrrc32 | Ikzf2 |
| 127 | 4.698E-54 | 1.993 | 0.824 | 0.282 | 1.184E-49 | CD4_Treg_Act-Lrrc32 | Sell |
| 128 | 2.461E-52 | 2.617 | 0.764 | 0.268 | 6.202E-48 | CD4_Treg_Act-Lrrc32 | Phlpp1 |
| 129 | 5.977E-47 | 2.210 | 0.797 | 0.343 | 1.506E-42 | CD4_Treg_Act-Lrrc32 | Lrig1 |
| 130 | 5.131E-45 | 2.392 | 0.615 | 0.177 | 1.293E-40 | CD4_Treg_Act-Lrrc32 | St8sia6 |
| 131 | 5.707E-41 | 2.932 | 0.453 | 0.107 | 1.438E-36 | CD4_Treg_Act-Lrrc32 | Ppm1l |
| 132 | 4.797E-39 | 2.992 | 0.378 | 0.080 | 1.209E-34 | CD4_Treg_Act-Lrrc32 | Swap70 |
| 133 | 7.245E-38 | 2.389 | 0.453 | 0.114 | 1.826E-33 | CD4_Treg_Act-Lrrc32 | Ecm1 |
| 134 | 2.293E-36 | 2.572 | 0.385 | 0.087 | 5.778E-32 | CD4_Treg_Act-Lrrc32 | Ikzf4 |
| 135 | 1.174E-33 | 2.758 | 0.392 | 0.096 | 2.958E-29 | CD4_Treg_Act-Lrrc32 | Ly6c1 |
| 136 | 3.610E-32 | 2.025 | 0.574 | 0.205 | 9.097E-28 | CD4_Treg_Act-Lrrc32 | Izumo1r |
| 137 | 2.550E-31 | 2.314 | 0.358 | 0.085 | 6.425E-27 | CD4_Treg_Act-Lrrc32 | Zbtb10 |
| 138 | 1.296E-27 | 2.103 | 0.649 | 0.307 | 3.266E-23 | CD4_Treg_Act-Lrrc32 | Aven |
| 139 | 3.552E-26 | 2.702 | 0.338 | 0.089 | 8.951E-22 | CD4_Treg_Act-Lrrc32 | Pip5k1b |
| 140 | 1.990E-09 | 2.130 | 0.250 | 0.104 | 5.016E-05 | CD4_Treg_Act-Lrrc32 | Ypel2 |
| 141 | 0.000E+00 | 5.219 | 0.799 | 0.034 | 0.000E+00 | CD4_Treg-Il1rl1 | Foxp3 |
| 142 | 1.463E-207 | 4.093 | 0.331 | 0.020 | 3.686E-203 | CD4_Treg-Il1rl1 | Lrrc32 |
| 143 | 2.826E-189 | 2.937 | 0.729 | 0.185 | 7.123E-185 | CD4_Treg-Il1rl1 | Il2ra |
| 144 | 8.034E-177 | 3.383 | 0.581 | 0.115 | 2.025E-172 | CD4_Treg-Il1rl1 | Neb |
| 145 | 2.335E-176 | 3.410 | 0.801 | 0.291 | 5.883E-172 | CD4_Treg-Il1rl1 | Ikzf2 |
| 146 | 2.666E-171 | 4.412 | 0.314 | 0.025 | 6.719E-167 | CD4_Treg-Il1rl1 | Ankrd55 |
| 147 | 1.011E-161 | 4.875 | 0.258 | 0.016 | 2.548E-157 | CD4_Treg-Il1rl1 | Klrg1 |
| 148 | 6.920E-160 | 3.239 | 0.636 | 0.163 | 1.744E-155 | CD4_Treg-Il1rl1 | Tnfrsf9 |
| 149 | 7.781E-160 | 2.959 | 0.839 | 0.367 | 1.961E-155 | CD4_Treg-Il1rl1 | Itgav |
| 150 | 5.349E-155 | 3.004 | 0.557 | 0.117 | 1.348E-150 | CD4_Treg-Il1rl1 | Dst |
| 151 | 7.151E-148 | 4.144 | 0.324 | 0.035 | 1.802E-143 | CD4_Treg-Il1rl1 | Il1rl1 |
| 152 | 5.147E-136 | 3.042 | 0.436 | 0.077 | 1.297E-131 | CD4_Treg-Il1rl1 | Gm13684 |
| 153 | 6.918E-126 | 2.865 | 0.485 | 0.103 | 1.743E-121 | CD4_Treg-Il1rl1 | Snx9 |
| 154 | 3.867E-102 | 2.762 | 0.451 | 0.109 | 9.745E-98 | CD4_Treg-Il1rl1 | Areg |
| 155 | 6.754E-102 | 2.804 | 0.383 | 0.078 | 1.702E-97 | CD4_Treg-Il1rl1 | Itgae |
| 156 | 7.536E-86 | 3.131 | 0.258 | 0.040 | 1.899E-81 | CD4_Treg-Il1rl1 | Ncmap |
| 157 | 2.273E-82 | 3.038 | 0.390 | 0.100 | 5.728E-78 | CD4_Treg-Il1rl1 | Lyn |
| 158 | 7.207E-81 | 2.872 | 0.275 | 0.048 | 1.816E-76 | CD4_Treg-Il1rl1 | Il10 |
| 159 | 3.469E-78 | 2.794 | 0.322 | 0.070 | 8.741E-74 | CD4_Treg-Il1rl1 | 2210408F21Rik |
| 160 | 2.523E-75 | 3.150 | 0.424 | 0.126 | 6.358E-71 | CD4_Treg-Il1rl1 | Myo3b |
| 161 | 1.423E-299 | 5.689 | 0.605 | 0.035 | 3.585E-295 | CD4_ISG-Mx1 | Mx1 |
| 162 | 6.385E-265 | 5.848 | 0.443 | 0.017 | 1.609E-260 | CD4_ISG-Mx1 | Ifit3b |
| 163 | 7.205E-241 | 5.232 | 0.680 | 0.065 | 1.816E-236 | CD4_ISG-Mx1 | Ifit3 |
| 164 | 2.753E-235 | 5.212 | 0.750 | 0.089 | 6.937E-231 | CD4_ISG-Mx1 | Ifit1 |
| 165 | 2.024E-216 | 4.902 | 0.649 | 0.067 | 5.099E-212 | CD4_ISG-Mx1 | Rsad2 |
| 166 | 1.182E-192 | 5.036 | 0.439 | 0.029 | 2.978E-188 | CD4_ISG-Mx1 | Mx2 |
| 167 | 8.820E-161 | 4.259 | 0.658 | 0.101 | 2.223E-156 | CD4_ISG-Mx1 | A330040F15Rik |
| 168 | 6.088E-154 | 3.707 | 0.592 | 0.077 | 1.534E-149 | CD4_ISG-Mx1 | Oas3 |
| 169 | 9.834E-147 | 3.897 | 0.588 | 0.081 | 2.478E-142 | CD4_ISG-Mx1 | Oasl2 |
| 170 | 1.050E-146 | 3.957 | 0.588 | 0.082 | 2.646E-142 | CD4_ISG-Mx1 | Usp18 |
| 171 | 7.023E-146 | 3.746 | 0.500 | 0.055 | 1.770E-141 | CD4_ISG-Mx1 | Cmpk2 |
| 172 | 4.324E-139 | 4.175 | 0.575 | 0.082 | 1.090E-134 | CD4_ISG-Mx1 | Ifit1bl1 |
| 173 | 1.125E-126 | 3.843 | 0.846 | 0.265 | 2.834E-122 | CD4_ISG-Mx1 | Isg15 |
| 174 | 2.563E-124 | 4.145 | 0.798 | 0.224 | 6.458E-120 | CD4_ISG-Mx1 | Slfn5 |
| 175 | 1.043E-121 | 3.523 | 0.719 | 0.166 | 2.630E-117 | CD4_ISG-Mx1 | Ifih1 |
| 176 | 4.968E-109 | 3.703 | 0.575 | 0.106 | 1.252E-104 | CD4_ISG-Mx1 | Ifit2 |
| 177 | 6.687E-99 | 4.548 | 0.373 | 0.046 | 1.685E-94 | CD4_ISG-Mx1 | Ifi204 |
| 178 | 6.629E-87 | 3.697 | 0.351 | 0.045 | 1.670E-82 | CD4_ISG-Mx1 | Phf11a |
| 179 | 1.511E-53 | 3.802 | 0.465 | 0.128 | 3.808E-49 | CD4_ISG-Mx1 | Cxcl10 |
| 180 | 6.901E-51 | 3.839 | 0.263 | 0.043 | 1.739E-46 | CD4_ISG-Mx1 | Ifi211 |
| 181 | 1.303E-124 | 3.627 | 0.495 | 0.062 | 3.283E-120 | CD4_Prolif-Ube2c | 1500009L16Rik |
| 182 | 1.606E-82 | 3.890 | 0.252 | 0.022 | 4.046E-78 | CD4_Prolif-Ube2c | Ube2c |
| 183 | 5.797E-80 | 2.855 | 0.294 | 0.031 | 1.461E-75 | CD4_Prolif-Ube2c | Nhsl2 |
| 184 | 1.256E-66 | 1.871 | 0.982 | 0.635 | 3.166E-62 | CD4_Prolif-Ube2c | Lgals1 |
| 185 | 7.586E-66 | 2.486 | 0.486 | 0.104 | 1.912E-61 | CD4_Prolif-Ube2c | Rap1gap2 |
| 186 | 2.159E-65 | 2.671 | 0.413 | 0.078 | 5.440E-61 | CD4_Prolif-Ube2c | Mmp25 |
| 187 | 1.594E-59 | 2.206 | 0.674 | 0.224 | 4.017E-55 | CD4_Prolif-Ube2c | Acsbg1 |
| 188 | 3.056E-56 | 2.537 | 0.353 | 0.066 | 7.702E-52 | CD4_Prolif-Ube2c | 1700012B07Rik |
| 189 | 1.278E-47 | 2.285 | 0.445 | 0.117 | 3.221E-43 | CD4_Prolif-Ube2c | Klf12 |
| 190 | 4.104E-40 | 1.834 | 0.720 | 0.335 | 1.034E-35 | CD4_Prolif-Ube2c | Lgals3 |
| 191 | 1.524E-39 | 2.093 | 0.284 | 0.058 | 3.840E-35 | CD4_Prolif-Ube2c | Il17re |
| 192 | 4.415E-39 | 1.814 | 0.482 | 0.151 | 1.113E-34 | CD4_Prolif-Ube2c | Tnfrsf25 |
| 193 | 1.001E-37 | 1.822 | 0.431 | 0.125 | 2.522E-33 | CD4_Prolif-Ube2c | Ltb4r1 |
| 194 | 1.290E-37 | 1.876 | 0.440 | 0.134 | 3.250E-33 | CD4_Prolif-Ube2c | Adam8 |
| 195 | 1.030E-35 | 1.895 | 0.376 | 0.103 | 2.596E-31 | CD4_Prolif-Ube2c | Serpinb1a |
| 196 | 4.409E-35 | 2.243 | 0.303 | 0.071 | 1.111E-30 | CD4_Prolif-Ube2c | E2f2 |
| 197 | 3.942E-34 | 2.011 | 0.275 | 0.060 | 9.935E-30 | CD4_Prolif-Ube2c | Gm17745 |
| 198 | 8.455E-32 | 1.901 | 0.362 | 0.105 | 2.131E-27 | CD4_Prolif-Ube2c | Aqp3 |
| 199 | 4.040E-27 | 2.058 | 0.321 | 0.097 | 1.018E-22 | CD4_Prolif-Ube2c | Ccr6 |
| 200 | 1.481E-25 | 1.792 | 0.271 | 0.073 | 3.733E-21 | CD4_Prolif-Ube2c | Fam124b |
| 201 | 2.513E-93 | 4.843 | 0.337 | 0.036 | 6.332E-89 | CD4_Str-Hspa1a | Atf5 |
| 202 | 2.475E-53 | 2.293 | 0.824 | 0.472 | 6.236E-49 | CD4_Str-Hspa1a | Bcl2 |
| 203 | 1.755E-43 | 3.401 | 0.387 | 0.097 | 4.424E-39 | CD4_Str-Hspa1a | Hspa1b |
| 204 | 3.382E-40 | 2.234 | 0.794 | 0.664 | 8.521E-36 | CD4_Str-Hspa1a | 2410006H16Rik |
| 205 | 5.377E-40 | 3.832 | 0.296 | 0.063 | 1.355E-35 | CD4_Str-Hspa1a | Hspa1a |
| 206 | 5.222E-37 | 2.169 | 0.744 | 0.466 | 1.316E-32 | CD4_Str-Hspa1a | Cdk6 |
| 207 | 1.583E-35 | 1.746 | 0.864 | 0.789 | 3.990E-31 | CD4_Str-Hspa1a | Klf6 |
| 208 | 2.382E-29 | 2.349 | 0.899 | 0.771 | 6.002E-25 | CD4_Str-Hspa1a | Scgb1a1 |
| 209 | 2.334E-28 | 1.958 | 0.633 | 0.387 | 5.880E-24 | CD4_Str-Hspa1a | Rcc2 |
| 210 | 2.310E-21 | 2.312 | 0.412 | 0.190 | 5.821E-17 | CD4_Str-Hspa1a | Slc7a5 |
| 211 | 4.718E-17 | 2.142 | 0.327 | 0.139 | 1.189E-12 | CD4_Str-Hspa1a | Aars |
| 212 | 1.889E-16 | 1.959 | 0.372 | 0.178 | 4.760E-12 | CD4_Str-Hspa1a | Iars |
| 213 | 1.389E-15 | 1.732 | 0.528 | 0.363 | 3.502E-11 | CD4_Str-Hspa1a | Snhg12 |
| 214 | 1.829E-15 | 2.193 | 0.296 | 0.125 | 4.610E-11 | CD4_Str-Hspa1a | Bbc3 |
| 215 | 2.975E-13 | 1.777 | 0.457 | 0.275 | 7.496E-09 | CD4_Str-Hspa1a | Cdkn1a |
| 216 | 7.613E-13 | 2.215 | 0.291 | 0.138 | 1.918E-08 | CD4_Str-Hspa1a | Xpot |
| 217 | 4.329E-11 | 1.706 | 0.362 | 0.205 | 1.091E-06 | CD4_Str-Hspa1a | Socs2 |
| 218 | 2.478E-10 | 1.783 | 0.357 | 0.218 | 6.245E-06 | CD4_Str-Hspa1a | Cebpg |
| 219 | 4.405E-08 | 1.772 | 0.533 | 0.441 | 1.110E-03 | CD4_Str-Hspa1a | Egr1 |
| 220 | 9.547E-08 | 1.743 | 0.261 | 0.148 | 2.406E-03 | CD4_Str-Hspa1a | 1110038B12Rik |

**Supplementary Table S3.** Top 20 marker genes for CD8⁺ T cell subclusters identified by lung scRNA-seq analysis.

|  | **p_value** | **avg_log2FC** | **pct.1** | **pct.2** | **p_value_adj** | **cluster** | **gene** |
| --- | --- | --- | --- | --- | --- | --- | --- |
| 1 | 1.495E-183 | 4.430 | 0.583 | 0.049 | 3.767E-179 | CD8_Naïve-Ccr7 | Sell |
| 2 | 7.537E-175 | 4.234 | 0.417 | 0.018 | 1.899E-170 | CD8_Naïve-Ccr7 | Nsg2 |
| 3 | 6.909E-136 | 2.839 | 0.800 | 0.153 | 1.741E-131 | CD8_Naïve-Ccr7 | Aff3 |
| 4 | 1.299E-124 | 4.362 | 0.365 | 0.024 | 3.273E-120 | CD8_Naïve-Ccr7 | Ccr7 |
| 5 | 5.211E-121 | 3.635 | 0.417 | 0.036 | 1.313E-116 | CD8_Naïve-Ccr7 | Patj |
| 6 | 3.788E-115 | 3.402 | 0.409 | 0.036 | 9.545E-111 | CD8_Naïve-Ccr7 | Ifngas1 |
| 7 | 6.919E-99 | 3.380 | 0.304 | 0.021 | 1.744E-94 | CD8_Naïve-Ccr7 | Treml2 |
| 8 | 1.740E-97 | 3.917 | 0.291 | 0.020 | 4.384E-93 | CD8_Naïve-Ccr7 | St6gal1 |
| 9 | 5.106E-97 | 4.196 | 0.252 | 0.013 | 1.287E-92 | CD8_Naïve-Ccr7 | Rflnb |
| 10 | 3.904E-96 | 3.816 | 0.291 | 0.020 | 9.838E-92 | CD8_Naïve-Ccr7 | Pakap.1 |
| 11 | 9.791E-96 | 2.769 | 0.483 | 0.066 | 2.467E-91 | CD8_Naïve-Ccr7 | Irs2 |
| 12 | 1.344E-85 | 2.225 | 0.804 | 0.243 | 3.386E-81 | CD8_Naïve-Ccr7 | Fgf13 |
| 13 | 3.197E-81 | 3.327 | 0.261 | 0.019 | 8.058E-77 | CD8_Naïve-Ccr7 | Tnfsf8 |
| 14 | 2.378E-79 | 2.857 | 0.391 | 0.053 | 5.992E-75 | CD8_Naïve-Ccr7 | Cd2ap |
| 15 | 3.792E-79 | 3.497 | 0.265 | 0.021 | 9.556E-75 | CD8_Naïve-Ccr7 | Zbtb10 |
| 16 | 2.724E-59 | 2.252 | 0.500 | 0.117 | 6.865E-55 | CD8_Naïve-Ccr7 | Bend4 |
| 17 | 4.021E-53 | 2.343 | 0.348 | 0.061 | 1.013E-48 | CD8_Naïve-Ccr7 | Nedd4l |
| 18 | 1.174E-51 | 2.441 | 0.326 | 0.055 | 2.959E-47 | CD8_Naïve-Ccr7 | Tnfrsf26 |
| 19 | 3.323E-47 | 2.226 | 0.326 | 0.059 | 8.373E-43 | CD8_Naïve-Ccr7 | Ttc28 |
| 20 | 2.860E-36 | 2.660 | 0.257 | 0.048 | 7.206E-32 | CD8_Naïve-Ccr7 | Acvr2a |
| 21 | 1.881E-43 | 1.848 | 0.839 | 0.505 | 4.741E-39 | CD8_EarlyAct-Ier3 | Gadd45b |
| 22 | 3.900E-31 | 1.911 | 0.693 | 0.396 | 9.828E-27 | CD8_EarlyAct-Ier3 | Tex14 |
| 23 | 6.874E-30 | 1.682 | 0.654 | 0.346 | 1.732E-25 | CD8_EarlyAct-Ier3 | Nfkbid |
| 24 | 3.465E-28 | 1.576 | 0.732 | 0.448 | 8.731E-24 | CD8_EarlyAct-Ier3 | Zfp36 |
| 25 | 9.481E-28 | 1.248 | 0.912 | 0.710 | 2.389E-23 | CD8_EarlyAct-Ier3 | Nfkbia |
| 26 | 4.691E-24 | 1.513 | 0.785 | 0.556 | 1.182E-19 | CD8_EarlyAct-Ier3 | Ifng |
| 27 | 3.213E-19 | 1.363 | 0.707 | 0.482 | 8.096E-15 | CD8_EarlyAct-Ier3 | Dhx40 |
| 28 | 3.980E-16 | 1.387 | 0.678 | 0.459 | 1.003E-11 | CD8_EarlyAct-Ier3 | Egr1 |
| 29 | 9.340E-16 | 1.384 | 0.385 | 0.178 | 2.354E-11 | CD8_EarlyAct-Ier3 | Nup107 |
| 30 | 8.706E-15 | 2.180 | 0.288 | 0.118 | 2.194E-10 | CD8_EarlyAct-Ier3 | Ier3 |
| 31 | 1.972E-14 | 1.246 | 0.595 | 0.417 | 4.970E-10 | CD8_EarlyAct-Ier3 | Ppp1r10 |
| 32 | 7.195E-14 | 1.357 | 0.634 | 0.477 | 1.813E-09 | CD8_EarlyAct-Ier3 | Icam1 |
| 33 | 1.747E-12 | 1.337 | 0.512 | 0.319 | 4.402E-08 | CD8_EarlyAct-Ier3 | Jun |
| 34 | 1.818E-12 | 1.710 | 0.341 | 0.173 | 4.581E-08 | CD8_EarlyAct-Ier3 | Tnf |
| 35 | 2.663E-11 | 1.509 | 0.298 | 0.142 | 6.712E-07 | CD8_EarlyAct-Ier3 | Kif23 |
| 36 | 8.549E-11 | 1.417 | 0.337 | 0.179 | 2.154E-06 | CD8_EarlyAct-Ier3 | Tmem230 |
| 37 | 2.666E-10 | 1.313 | 0.371 | 0.210 | 6.719E-06 | CD8_EarlyAct-Ier3 | Slc7a5 |
| 38 | 1.340E-08 | 1.131 | 0.468 | 0.328 | 3.377E-04 | CD8_EarlyAct-Ier3 | Snhg12 |
| 39 | 2.911E-08 | 1.494 | 0.268 | 0.143 | 7.336E-04 | CD8_EarlyAct-Ier3 | Snhg9 |
| 40 | 9.324E-08 | 1.168 | 0.356 | 0.218 | 2.350E-03 | CD8_EarlyAct-Ier3 | Tfrc |
| 41 | 1.190E-216 | 5.086 | 0.788 | 0.072 | 3.000E-212 | CD8_ISG-Ifit1 | Ifit1 |
| 42 | 9.989E-184 | 5.068 | 0.737 | 0.077 | 2.517E-179 | CD8_ISG-Ifit1 | Ifit3 |
| 43 | 2.447E-159 | 5.157 | 0.447 | 0.024 | 6.165E-155 | CD8_ISG-Ifit1 | Ifit3b |
| 44 | 2.565E-139 | 3.840 | 0.760 | 0.118 | 6.465E-135 | CD8_ISG-Ifit1 | Gbp6 |
| 45 | 2.087E-135 | 4.670 | 0.542 | 0.051 | 5.260E-131 | CD8_ISG-Ifit1 | Rsad2 |
| 46 | 1.893E-121 | 4.047 | 0.464 | 0.039 | 4.771E-117 | CD8_ISG-Ifit1 | Cmpk2 |
| 47 | 9.405E-113 | 3.265 | 0.737 | 0.137 | 2.370E-108 | CD8_ISG-Ifit1 | Rtp4 |
| 48 | 1.842E-112 | 3.775 | 0.620 | 0.090 | 4.642E-108 | CD8_ISG-Ifit1 | Mx1 |
| 49 | 9.199E-109 | 3.927 | 0.581 | 0.079 | 2.318E-104 | CD8_ISG-Ifit1 | Usp18 |
| 50 | 4.426E-99 | 3.535 | 0.659 | 0.121 | 1.115E-94 | CD8_ISG-Ifit1 | Isg20 |
| 51 | 5.819E-99 | 4.265 | 0.497 | 0.062 | 1.466E-94 | CD8_ISG-Ifit1 | Slfn5 |
| 52 | 3.945E-95 | 3.176 | 0.620 | 0.105 | 9.941E-91 | CD8_ISG-Ifit1 | Phf11c |
| 53 | 6.127E-95 | 3.536 | 0.654 | 0.122 | 1.544E-90 | CD8_ISG-Ifit1 | Ifit1bl1 |
| 54 | 7.927E-88 | 4.725 | 0.291 | 0.019 | 1.998E-83 | CD8_ISG-Ifit1 | Mx2 |
| 55 | 3.595E-82 | 4.807 | 0.341 | 0.032 | 9.060E-78 | CD8_ISG-Ifit1 | Iigp1 |
| 56 | 8.953E-79 | 3.045 | 0.810 | 0.257 | 2.256E-74 | CD8_ISG-Ifit1 | Herc6 |
| 57 | 3.604E-74 | 3.300 | 0.816 | 0.304 | 9.083E-70 | CD8_ISG-Ifit1 | Isg15 |
| 58 | 1.380E-52 | 3.087 | 0.497 | 0.115 | 3.479E-48 | CD8_ISG-Ifit1 | Ifit2 |
| 59 | 5.807E-49 | 3.566 | 0.279 | 0.037 | 1.463E-44 | CD8_ISG-Ifit1 | Oasl2 |
| 60 | 1.161E-41 | 3.598 | 0.408 | 0.095 | 2.925E-37 | CD8_ISG-Ifit1 | Cxcl10 |
| 61 | 8.161E-157 | 5.855 | 0.600 | 0.011 | 2.056E-152 | CD8_Prolif-Mki67 | Pclaf |
| 62 | 8.011E-147 | 5.818 | 0.486 | 0.007 | 2.019E-142 | CD8_Prolif-Mki67 | Cdca3 |
| 63 | 1.462E-142 | 5.123 | 0.743 | 0.021 | 3.684E-138 | CD8_Prolif-Mki67 | Ube2c |
| 64 | 1.418E-131 | 6.054 | 0.629 | 0.016 | 3.574E-127 | CD8_Prolif-Mki67 | Ccnb2 |
| 65 | 7.235E-126 | 5.948 | 0.600 | 0.016 | 1.823E-121 | CD8_Prolif-Mki67 | Birc5 |
| 66 | 1.181E-124 | 5.762 | 0.457 | 0.008 | 2.975E-120 | CD8_Prolif-Mki67 | Cdkn3 |
| 67 | 4.459E-93 | 4.812 | 0.714 | 0.034 | 1.124E-88 | CD8_Prolif-Mki67 | Cks1b |
| 68 | 2.506E-53 | 3.722 | 0.514 | 0.030 | 6.314E-49 | CD8_Prolif-Mki67 | Knstrn |
| 69 | 5.790E-44 | 4.574 | 0.257 | 0.009 | 1.459E-39 | CD8_Prolif-Mki67 | Cenpm |
| 70 | 5.577E-36 | 3.286 | 0.714 | 0.088 | 1.405E-31 | CD8_Prolif-Mki67 | Mki67 |
| 71 | 8.491E-33 | 3.101 | 0.343 | 0.022 | 2.140E-28 | CD8_Prolif-Mki67 | Cenpe |
| 72 | 2.430E-27 | 3.791 | 0.343 | 0.027 | 6.123E-23 | CD8_Prolif-Mki67 | Cdca8 |
| 73 | 5.600E-26 | 3.241 | 0.857 | 0.216 | 1.411E-21 | CD8_Prolif-Mki67 | Hmgn2 |
| 74 | 7.661E-26 | 3.333 | 0.286 | 0.020 | 1.931E-21 | CD8_Prolif-Mki67 | Cep55 |
| 75 | 8.151E-25 | 2.859 | 0.943 | 0.268 | 2.054E-20 | CD8_Prolif-Mki67 | Stmn1 |
| 76 | 1.609E-23 | 2.973 | 0.371 | 0.036 | 4.054E-19 | CD8_Prolif-Mki67 | Hmgb3 |
| 77 | 5.947E-21 | 2.782 | 1.000 | 0.828 | 1.499E-16 | CD8_Prolif-Mki67 | Hmgb2 |
| 78 | 2.296E-20 | 2.788 | 0.400 | 0.048 | 5.786E-16 | CD8_Prolif-Mki67 | H2bc4 |
| 79 | 7.481E-20 | 2.762 | 0.743 | 0.178 | 1.885E-15 | CD8_Prolif-Mki67 | Cenpa |
| 80 | 3.609E-18 | 2.655 | 0.257 | 0.023 | 9.094E-14 | CD8_Prolif-Mki67 | Mylpf |
| 81 | 4.265E-67 | 1.012 | 0.956 | 0.883 | 1.075E-62 | CD8_Tem-Grap2 | Arhgap26 |
| 82 | 4.316E-59 | 1.100 | 0.947 | 0.872 | 1.088E-54 | CD8_Tem-Grap2 | Grap2 |
| 83 | 1.869E-56 | 0.986 | 0.934 | 0.795 | 4.710E-52 | CD8_Tem-Grap2 | Themis |
| 84 | 7.231E-32 | 1.067 | 0.755 | 0.630 | 1.822E-27 | CD8_Tem-Grap2 | Atp10d |
| 85 | 3.888E-19 | 1.011 | 0.645 | 0.551 | 9.797E-15 | CD8_Tem-Grap2 | Pde4d |
| 86 | 5.509E-18 | 1.248 | 0.465 | 0.318 | 1.388E-13 | CD8_Tem-Grap2 | Lamc1 |
| 87 | 1.347E-16 | 1.448 | 0.304 | 0.160 | 3.395E-12 | CD8_Tem-Grap2 | Plekha6 |
| 88 | 4.821E-16 | 1.320 | 0.366 | 0.223 | 1.215E-11 | CD8_Tem-Grap2 | Pde8a |
| 89 | 5.003E-15 | 1.393 | 0.332 | 0.203 | 1.261E-10 | CD8_Tem-Grap2 | Arhgef12 |
| 90 | 4.110E-14 | 1.072 | 0.491 | 0.373 | 1.036E-09 | CD8_Tem-Grap2 | Notch2 |
| 91 | 2.065E-13 | 1.070 | 0.556 | 0.463 | 5.205E-09 | CD8_Tem-Grap2 | Marchf3 |
| 92 | 3.567E-13 | 1.195 | 0.442 | 0.323 | 8.990E-09 | CD8_Tem-Grap2 | Xylt1 |
| 93 | 3.426E-09 | 1.164 | 0.408 | 0.329 | 8.634E-05 | CD8_Tem-Grap2 | St6galnac3 |
| 94 | 1.256E-08 | 1.071 | 0.250 | 0.158 | 3.166E-04 | CD8_Tem-Grap2 | Rev1 |
| 95 | 2.457E-07 | 1.043 | 0.427 | 0.354 | 6.193E-03 | CD8_Tem-Grap2 | Herc3 |
| 96 | 2.969E-07 | 0.994 | 0.414 | 0.345 | 7.483E-03 | CD8_Tem-Grap2 | Exoc6b |
| 97 | 3.012E-07 | 0.990 | 0.252 | 0.168 | 7.590E-03 | CD8_Tem-Grap2 | Dcp1b |
| 98 | 3.866E-07 | 1.152 | 0.431 | 0.362 | 9.743E-03 | CD8_Tem-Grap2 | Insyn2b |
| 99 | 6.310E-07 | 1.114 | 0.256 | 0.179 | 1.590E-02 | CD8_Tem-Grap2 | Diaph3 |
| 100 | 1.841E-06 | 1.009 | 0.313 | 0.241 | 4.638E-02 | CD8_Tem-Grap2 | Smurf1 |
| 101 | 5.617E-58 | 0.927 | 0.981 | 0.876 | 1.416E-53 | CD8_Tem-S1pr5 | Lgals1 |
| 102 | 2.025E-52 | 0.717 | 0.998 | 0.980 | 5.102E-48 | CD8_Tem-S1pr5 | Ccl5 |
| 103 | 6.863E-37 | 0.744 | 0.902 | 0.785 | 1.730E-32 | CD8_Tem-S1pr5 | Emp3 |
| 104 | 1.106E-34 | 0.849 | 0.926 | 0.788 | 2.786E-30 | CD8_Tem-S1pr5 | Klf2 |
| 105 | 7.585E-31 | 0.673 | 0.919 | 0.862 | 1.912E-26 | CD8_Tem-S1pr5 | Cyba |
| 106 | 1.375E-30 | 1.653 | 0.371 | 0.156 | 3.466E-26 | CD8_Tem-S1pr5 | Actn2 |
| 107 | 2.664E-26 | 0.996 | 0.548 | 0.303 | 6.712E-22 | CD8_Tem-S1pr5 | S1pr5 |
| 108 | 4.429E-23 | 0.654 | 0.849 | 0.742 | 1.116E-18 | CD8_Tem-S1pr5 | S100a13 |
| 109 | 1.304E-21 | 1.319 | 0.409 | 0.221 | 3.286E-17 | CD8_Tem-S1pr5 | Dtx1 |
| 110 | 8.348E-21 | 0.736 | 0.725 | 0.529 | 2.104E-16 | CD8_Tem-S1pr5 | Cx3cr1 |
| 111 | 1.033E-19 | 0.680 | 0.708 | 0.536 | 2.604E-15 | CD8_Tem-S1pr5 | Rasgrp2 |
| 112 | 2.221E-16 | 0.759 | 0.548 | 0.364 | 5.596E-12 | CD8_Tem-S1pr5 | Klrg1 |
| 113 | 1.575E-14 | 0.685 | 0.505 | 0.319 | 3.969E-10 | CD8_Tem-S1pr5 | St6galnac3 |
| 114 | 3.400E-11 | 0.934 | 0.641 | 0.559 | 8.568E-07 | CD8_Tem-S1pr5 | Ifng |
| 115 | 5.468E-11 | 0.670 | 0.577 | 0.480 | 1.378E-06 | CD8_Tem-S1pr5 | S1pr4 |
| 116 | 8.724E-10 | 0.845 | 0.356 | 0.233 | 2.198E-05 | CD8_Tem-S1pr5 | Kcnj8 |
| 117 | 6.635E-09 | 0.734 | 0.500 | 0.427 | 1.672E-04 | CD8_Tem-S1pr5 | Klk8 |
| 118 | 4.204E-08 | 0.753 | 0.443 | 0.351 | 1.059E-03 | CD8_Tem-S1pr5 | Nipal3 |
| 119 | 3.712E-07 | 0.856 | 0.278 | 0.184 | 9.355E-03 | CD8_Tem-S1pr5 | Borcs7 |
| 120 | 5.342E-07 | 0.717 | 0.368 | 0.288 | 1.346E-02 | CD8_Tem-S1pr5 | Ccdc85b |
| 121 | 2.003E-43 | 0.778 | 0.900 | 0.583 | 5.048E-39 | CD8_Teff-Klrg1 | Cd8b1 |
| 122 | 4.125E-35 | 0.979 | 0.634 | 0.345 | 1.039E-30 | CD8_Teff-Klrg1 | Klrg1 |
| 123 | 4.488E-34 | 0.844 | 0.706 | 0.432 | 1.131E-29 | CD8_Teff-Klrg1 | Prf1 |
| 124 | 1.362E-29 | 1.084 | 0.456 | 0.214 | 3.432E-25 | CD8_Teff-Klrg1 | Kcnj8 |
| 125 | 5.372E-28 | 0.789 | 0.726 | 0.497 | 1.354E-23 | CD8_Teff-Klrg1 | Klrc1 |
| 126 | 1.361E-27 | 0.814 | 0.562 | 0.295 | 3.430E-23 | CD8_Teff-Klrg1 | S1pr5 |
| 127 | 1.437E-25 | 1.385 | 0.358 | 0.167 | 3.621E-21 | CD8_Teff-Klrg1 | Med12l |
| 128 | 3.849E-24 | 1.099 | 0.352 | 0.158 | 9.701E-20 | CD8_Teff-Klrg1 | As3mt |
| 129 | 9.726E-19 | 1.180 | 0.284 | 0.133 | 2.451E-14 | CD8_Teff-Klrg1 | H2-Q10 |
| 130 | 1.155E-15 | 0.898 | 0.313 | 0.161 | 2.911E-11 | CD8_Teff-Klrg1 | Plekha6 |
| 131 | 1.843E-15 | 1.058 | 0.286 | 0.145 | 4.644E-11 | CD8_Teff-Klrg1 | Smpdl3b |
| 132 | 4.861E-15 | 0.876 | 0.364 | 0.212 | 1.225E-10 | CD8_Teff-Klrg1 | Entpd1 |
| 133 | 1.060E-14 | 0.792 | 0.399 | 0.233 | 2.671E-10 | CD8_Teff-Klrg1 | Pogk |
| 134 | 2.317E-14 | 0.905 | 0.305 | 0.164 | 5.838E-10 | CD8_Teff-Klrg1 | Eomes |
| 135 | 2.545E-13 | 0.812 | 0.315 | 0.175 | 6.413E-09 | CD8_Teff-Klrg1 | Camk2n1 |
| 136 | 5.807E-13 | 0.923 | 0.311 | 0.176 | 1.463E-08 | CD8_Teff-Klrg1 | Borcs7 |
| 137 | 1.557E-10 | 0.924 | 0.252 | 0.140 | 3.924E-06 | CD8_Teff-Klrg1 | Lair1 |
| 138 | 7.954E-09 | 0.772 | 0.305 | 0.197 | 2.004E-04 | CD8_Teff-Klrg1 | Chpt1 |
| 139 | 6.317E-07 | 0.838 | 0.270 | 0.185 | 1.592E-02 | CD8_Teff-Klrg1 | Wdr95 |
| 140 | 6.408E-07 | 0.768 | 0.258 | 0.172 | 1.615E-02 | CD8_Teff-Klrg1 | Lrp6 |
| 141 | 8.188E-150 | 2.291 | 0.613 | 0.153 | 2.063E-145 | CD8_Trm-Itgae | Ctla4 |
| 142 | 3.715E-144 | 2.108 | 0.833 | 0.369 | 9.362E-140 | CD8_Trm-Itgae | Ctla2a |
| 143 | 7.698E-129 | 1.761 | 0.861 | 0.423 | 1.940E-124 | CD8_Trm-Itgae | Ltb |
| 144 | 2.108E-124 | 1.522 | 0.787 | 0.330 | 5.312E-120 | CD8_Trm-Itgae | Ly6a |
| 145 | 2.063E-123 | 2.243 | 0.525 | 0.125 | 5.198E-119 | CD8_Trm-Itgae | Cxcr3 |
| 146 | 5.630E-100 | 1.493 | 0.792 | 0.400 | 1.419E-95 | CD8_Trm-Itgae | Inpp4b |
| 147 | 3.357E-86 | 2.069 | 0.403 | 0.098 | 8.460E-82 | CD8_Trm-Itgae | Rgs10 |
| 148 | 5.148E-80 | 1.721 | 0.592 | 0.240 | 1.297E-75 | CD8_Trm-Itgae | Cxcr6 |
| 149 | 1.366E-78 | 1.696 | 0.544 | 0.200 | 3.443E-74 | CD8_Trm-Itgae | Gpr183 |
| 150 | 1.720E-78 | 2.621 | 0.279 | 0.048 | 4.334E-74 | CD8_Trm-Itgae | Pdcd1 |
| 151 | 4.633E-71 | 2.925 | 0.261 | 0.049 | 1.168E-66 | CD8_Trm-Itgae | Itgae |
| 152 | 4.844E-71 | 1.940 | 0.390 | 0.112 | 1.221E-66 | CD8_Trm-Itgae | Ctla2b |
| 153 | 4.865E-64 | 1.498 | 0.457 | 0.161 | 1.226E-59 | CD8_Trm-Itgae | Pmepa1 |
| 154 | 1.570E-63 | 2.148 | 0.307 | 0.075 | 3.957E-59 | CD8_Trm-Itgae | Igflr1 |
| 155 | 7.189E-51 | 1.425 | 0.722 | 0.493 | 1.812E-46 | CD8_Trm-Itgae | S100a4 |
| 156 | 9.190E-46 | 1.523 | 0.335 | 0.113 | 2.316E-41 | CD8_Trm-Itgae | Serpina3g |
| 157 | 4.320E-44 | 3.191 | 0.391 | 0.163 | 1.089E-39 | CD8_Trm-Itgae | Ifitm1 |
| 158 | 3.463E-39 | 1.459 | 0.295 | 0.102 | 8.727E-35 | CD8_Trm-Itgae | Gstp3 |
| 159 | 9.168E-31 | 2.114 | 0.271 | 0.105 | 2.310E-26 | CD8_Trm-Itgae | AA467197 |
| 160 | 5.113E-26 | 1.777 | 0.292 | 0.130 | 1.288E-21 | CD8_Trm-Itgae | Ifitm2 |
| 161 | 9.436E-91 | 7.281 | 0.562 | 0.050 | 2.378E-86 | CD8_Tex-Lag3 | Xcl1 |
| 162 | 7.948E-38 | 3.771 | 0.360 | 0.048 | 2.003E-33 | CD8_Tex-Lag3 | Rgs16 |
| 163 | 1.711E-37 | 3.129 | 0.663 | 0.178 | 4.311E-33 | CD8_Tex-Lag3 | Tnfrsf9 |
| 164 | 1.194E-32 | 2.769 | 0.494 | 0.103 | 3.009E-28 | CD8_Tex-Lag3 | Kcnq5 |
| 165 | 5.191E-32 | 2.253 | 0.461 | 0.085 | 1.308E-27 | CD8_Tex-Lag3 | Cables1 |
| 166 | 1.677E-31 | 2.943 | 0.461 | 0.092 | 4.226E-27 | CD8_Tex-Lag3 | Lag3 |
| 167 | 2.299E-27 | 2.178 | 0.764 | 0.289 | 5.795E-23 | CD8_Tex-Lag3 | Ikzf2 |
| 168 | 1.588E-25 | 2.227 | 0.483 | 0.113 | 4.003E-21 | CD8_Tex-Lag3 | Igflr1 |
| 169 | 4.568E-25 | 2.259 | 0.416 | 0.089 | 1.151E-20 | CD8_Tex-Lag3 | Klra6 |
| 170 | 3.788E-23 | 2.676 | 0.326 | 0.060 | 9.545E-19 | CD8_Tex-Lag3 | Pdcd1lg2 |
| 171 | 8.489E-23 | 2.409 | 0.292 | 0.049 | 2.139E-18 | CD8_Tex-Lag3 | Fndc3b |
| 172 | 8.976E-23 | 2.061 | 0.461 | 0.115 | 2.262E-18 | CD8_Tex-Lag3 | Lat2 |
| 173 | 2.328E-21 | 2.413 | 0.281 | 0.048 | 5.867E-17 | CD8_Tex-Lag3 | Smim36 |
| 174 | 1.455E-18 | 2.248 | 0.697 | 0.354 | 3.667E-14 | CD8_Tex-Lag3 | Cd7 |
| 175 | 1.509E-18 | 2.233 | 0.258 | 0.047 | 3.802E-14 | CD8_Tex-Lag3 | Sulf2 |
| 176 | 4.517E-18 | 2.076 | 0.326 | 0.073 | 1.138E-13 | CD8_Tex-Lag3 | Trgc4 |
| 177 | 4.073E-15 | 2.284 | 0.371 | 0.110 | 1.026E-10 | CD8_Tex-Lag3 | Irf8 |
| 178 | 9.039E-09 | 2.266 | 0.292 | 0.106 | 2.278E-04 | CD8_Tex-Lag3 | Il2ra |
| 179 | 1.967E-07 | 2.377 | 0.371 | 0.166 | 4.958E-03 | CD8_Tex-Lag3 | Crtam |
| 180 | 1.876E-06 | 2.090 | 0.315 | 0.139 | 4.728E-02 | CD8_Tex-Lag3 | Pkp4 |
| 181 | 0.000E+00 | 3.138 | 0.921 | 0.170 | 0.000E+00 | CD8_NKT-Klrb1c | Ikzf2 |
| 182 | 1.542E-267 | 3.876 | 0.682 | 0.090 | 3.885E-263 | CD8_NKT-Klrb1c | Myo1e |
| 183 | 7.594E-265 | 3.345 | 0.835 | 0.220 | 1.914E-260 | CD8_NKT-Klrb1c | Fcer1g |
| 184 | 4.569E-236 | 3.298 | 0.687 | 0.112 | 1.151E-231 | CD8_NKT-Klrb1c | Spry2 |
| 185 | 3.085E-222 | 3.112 | 0.705 | 0.135 | 7.774E-218 | CD8_NKT-Klrb1c | Klrb1c |
| 186 | 4.102E-206 | 4.531 | 0.442 | 0.031 | 1.034E-201 | CD8_NKT-Klrb1c | Clnk |
| 187 | 3.032E-202 | 4.186 | 0.426 | 0.027 | 7.641E-198 | CD8_NKT-Klrb1c | Klra6 |
| 188 | 2.607E-178 | 3.248 | 0.597 | 0.115 | 6.571E-174 | CD8_NKT-Klrb1c | Cnnm2 |
| 189 | 1.600E-177 | 3.060 | 0.584 | 0.104 | 4.031E-173 | CD8_NKT-Klrb1c | Klra9 |
| 190 | 1.905E-168 | 3.174 | 0.543 | 0.094 | 4.801E-164 | CD8_NKT-Klrb1c | Cd244a |
| 191 | 5.490E-153 | 3.080 | 0.543 | 0.107 | 1.383E-148 | CD8_NKT-Klrb1c | Srgap3 |
| 192 | 1.653E-143 | 2.566 | 0.610 | 0.157 | 4.165E-139 | CD8_NKT-Klrb1c | Plcg2 |
| 193 | 2.460E-97 | 2.645 | 0.465 | 0.126 | 6.200E-93 | CD8_NKT-Klrb1c | Itga2 |
| 194 | 7.867E-97 | 3.537 | 0.288 | 0.038 | 1.982E-92 | CD8_NKT-Klrb1c | Atp1b1 |
| 195 | 3.051E-85 | 2.756 | 0.354 | 0.075 | 7.688E-81 | CD8_NKT-Klrb1c | Lat2 |
| 196 | 4.071E-79 | 2.698 | 0.337 | 0.072 | 1.026E-74 | CD8_NKT-Klrb1c | Gab2 |
| 197 | 3.318E-78 | 2.595 | 0.578 | 0.273 | 8.362E-74 | CD8_NKT-Klrb1c | Tyrobp |
| 198 | 1.013E-73 | 2.702 | 0.417 | 0.128 | 2.554E-69 | CD8_NKT-Klrb1c | Lyn |
| 199 | 1.077E-57 | 2.274 | 0.378 | 0.123 | 2.715E-53 | CD8_NKT-Klrb1c | Klf12 |
| 200 | 2.619E-47 | 2.447 | 0.273 | 0.079 | 6.600E-43 | CD8_NKT-Klrb1c | Itgam |

**Supplementary Table S4.** Top 20 marker genes for B cell subclusters identified by lung scRNA-seq analysis.

|  | **p_value** | **avg_log2FC** | **pct.1** | **pct.2** | **p_value_adj** | **cluster** | **gene** |
| --- | --- | --- | --- | --- | --- | --- | --- |
| 1 | 1.434E-287 | 6.487552618 | 0.511 | 0.018 | 3.614E-283 | B_Transitional-Sox4 | Agbl1 |
| 2 | 1.129E-160 | 4.667461648 | 0.459 | 0.038 | 2.846E-156 | B_Transitional-Sox4 | Nedd4 |
| 3 | 5.036E-154 | 4.166094548 | 0.506 | 0.05 | 1.269E-149 | B_Transitional-Sox4 | Niban3 |
| 4 | 2.613E-111 | 3.836539297 | 0.468 | 0.061 | 6.585E-107 | B_Transitional-Sox4 | Akap12 |
| 5 | 1.092E-87 | 2.564201524 | 0.853 | 0.309 | 2.751E-83 | B_Transitional-Sox4 | Cecr2 |
| 6 | 1.925E-73 | 3.151154498 | 0.576 | 0.149 | 4.851E-69 | B_Transitional-Sox4 | Klhl14 |
| 7 | 4.620E-72 | 2.486958423 | 0.701 | 0.24 | 1.164E-67 | B_Transitional-Sox4 | Mctp2 |
| 8 | 2.720E-70 | 2.446180339 | 0.823 | 0.389 | 6.855E-66 | B_Transitional-Sox4 | Spib |
| 9 | 2.461E-60 | 3.587253306 | 0.255 | 0.032 | 6.201E-56 | B_Transitional-Sox4 | Sox4 |
| 10 | 3.946E-60 | 2.393948045 | 0.736 | 0.304 | 9.944E-56 | B_Transitional-Sox4 | Hdac9 |
| 11 | 2.024E-57 | 2.352064241 | 0.589 | 0.178 | 5.100E-53 | B_Transitional-Sox4 | Hck |
| 12 | 3.326E-52 | 2.842088063 | 0.437 | 0.11 | 8.382E-48 | B_Transitional-Sox4 | Fhit |
| 13 | 6.515E-49 | 2.927552794 | 0.312 | 0.06 | 1.642E-44 | B_Transitional-Sox4 | Lynx1 |
| 14 | 2.089E-47 | 2.775631556 | 0.381 | 0.092 | 5.263E-43 | B_Transitional-Sox4 | Sox5 |
| 15 | 3.048E-44 | 2.591210864 | 0.351 | 0.081 | 7.682E-40 | B_Transitional-Sox4 | Eps8 |
| 16 | 2.422E-42 | 2.436261534 | 0.429 | 0.124 | 6.104E-38 | B_Transitional-Sox4 | Bcr |
| 17 | 3.018E-39 | 2.353590748 | 0.494 | 0.171 | 7.606E-35 | B_Transitional-Sox4 | Serinc5 |
| 18 | 4.289E-39 | 2.40688381 | 0.403 | 0.115 | 1.081E-34 | B_Transitional-Sox4 | Ppm1e |
| 19 | 5.681E-38 | 2.708962725 | 0.385 | 0.112 | 1.432E-33 | B_Transitional-Sox4 | Tifa |
| 20 | 1.098E-32 | 2.400325395 | 0.281 | 0.068 | 2.766E-28 | B_Transitional-Sox4 | Il12a |
| 21 | 1.168E-104 | 1.202161337 | 0.787 | 0.539 | 2.945E-100 | B_Naïve-like | Pgap1 |
| 22 | 1.335E-103 | 0.966824096 | 0.917 | 0.773 | 3.363E-99 | B_Naïve-like | Xylt1 |
| 23 | 8.283E-49 | 1.122974834 | 0.54 | 0.347 | 2.087E-44 | B_Naïve-like | Adamts6 |
| 24 | 4.885E-40 | 1.266079564 | 0.419 | 0.241 | 1.231E-35 | B_Naïve-like | Ssbp2 |
| 25 | 1.014E-37 | 0.965555964 | 0.518 | 0.334 | 2.555E-33 | B_Naïve-like | Zbtb10 |
| 26 | 2.611E-37 | 1.159172983 | 0.492 | 0.321 | 6.579E-33 | B_Naïve-like | Maml3 |
| 27 | 4.447E-32 | 0.952835257 | 0.546 | 0.412 | 1.121E-27 | B_Naïve-like | Rfx3 |
| 28 | 2.396E-26 | 1.017075081 | 0.528 | 0.413 | 6.037E-22 | B_Naïve-like | Antxr2 |
| 29 | 1.221E-24 | 1.016435859 | 0.354 | 0.22 | 3.076E-20 | B_Naïve-like | Rapgef4 |
| 30 | 5.911E-22 | 1.21035617 | 0.394 | 0.282 | 1.490E-17 | B_Naïve-like | Flnb |
| 31 | 8.858E-22 | 1.120532464 | 0.335 | 0.22 | 2.232E-17 | B_Naïve-like | Exoc6b |
| 32 | 4.617E-21 | 0.921164358 | 0.501 | 0.392 | 1.163E-16 | B_Naïve-like | Lamc1 |
| 33 | 4.275E-20 | 0.93138946 | 0.339 | 0.216 | 1.077E-15 | B_Naïve-like | Slc16a10 |
| 34 | 2.680E-18 | 1.100135476 | 0.283 | 0.178 | 6.753E-14 | B_Naïve-like | Tnik |
| 35 | 4.378E-18 | 0.943448366 | 0.327 | 0.214 | 1.103E-13 | B_Naïve-like | Patj |
| 36 | 1.039E-16 | 1.031964039 | 0.293 | 0.194 | 2.618E-12 | B_Naïve-like | Myo9a |
| 37 | 2.877E-16 | 0.934750802 | 0.272 | 0.173 | 7.251E-12 | B_Naïve-like | Gpr137c |
| 38 | 6.880E-16 | 1.03682537 | 0.322 | 0.225 | 1.734E-11 | B_Naïve-like | Gbe1 |
| 39 | 7.857E-13 | 0.931205828 | 0.301 | 0.22 | 1.980E-08 | B_Naïve-like | Pde8a |
| 40 | 3.215E-10 | 0.927041073 | 0.372 | 0.306 | 8.102E-06 | B_Naïve-like | Insyn2b |
| 41 | 1.945E-133 | 3.056382727 | 0.471 | 0.075 | 4.901E-129 | B_ISG-Ifit3 | Rtp4 |
| 42 | 2.211E-124 | 4.396408125 | 0.31 | 0.03 | 5.572E-120 | B_ISG-Ifit3 | Ifit3 |
| 43 | 1.137E-99 | 2.203840469 | 0.715 | 0.271 | 2.864E-95 | B_ISG-Ifit3 | Ifi206 |
| 44 | 1.287E-97 | 2.366156959 | 0.667 | 0.247 | 3.242E-93 | B_ISG-Ifit3 | Ifi213 |
| 45 | 1.647E-97 | 3.134771858 | 0.521 | 0.137 | 4.150E-93 | B_ISG-Ifit3 | Slfn5 |
| 46 | 1.245E-96 | 2.555582501 | 0.553 | 0.156 | 3.136E-92 | B_ISG-Ifit3 | Ifi47 |
| 47 | 1.761E-94 | 2.494236199 | 0.603 | 0.2 | 4.437E-90 | B_ISG-Ifit3 | Zbp1 |
| 48 | 2.578E-90 | 3.370960991 | 0.27 | 0.033 | 6.497E-86 | B_ISG-Ifit3 | Gbp6 |
| 49 | 6.648E-90 | 3.077382673 | 0.328 | 0.052 | 1.675E-85 | B_ISG-Ifit3 | Tgtp2 |
| 50 | 2.284E-85 | 3.285468175 | 0.31 | 0.049 | 5.755E-81 | B_ISG-Ifit3 | Usp18 |
| 51 | 3.732E-85 | 2.588290656 | 0.457 | 0.113 | 9.404E-81 | B_ISG-Ifit3 | Irf7 |
| 52 | 2.865E-81 | 2.874700459 | 0.288 | 0.043 | 7.219E-77 | B_ISG-Ifit3 | Oas2 |
| 53 | 9.576E-80 | 2.420948031 | 0.538 | 0.171 | 2.413E-75 | B_ISG-Ifit3 | Irgm1 |
| 54 | 6.754E-79 | 2.890054061 | 0.32 | 0.057 | 1.702E-74 | B_ISG-Ifit3 | Oasl2 |
| 55 | 5.136E-75 | 2.668034904 | 0.419 | 0.106 | 1.294E-70 | B_ISG-Ifit3 | Gbp7 |
| 56 | 1.411E-65 | 2.24289146 | 0.37 | 0.089 | 3.556E-61 | B_ISG-Ifit3 | Oasl1 |
| 57 | 3.154E-58 | 2.644142971 | 0.268 | 0.053 | 7.947E-54 | B_ISG-Ifit3 | Serpina3f |
| 58 | 6.215E-50 | 2.32149067 | 0.305 | 0.078 | 1.566E-45 | B_ISG-Ifit3 | Irgm2 |
| 59 | 1.282E-48 | 2.305495455 | 0.352 | 0.108 | 3.230E-44 | B_ISG-Ifit3 | Xaf1 |
| 60 | 2.904E-34 | 2.29380166 | 0.308 | 0.106 | 7.317E-30 | B_ISG-Ifit3 | Socs1 |
| 61 | 2.414E-101 | 1.163918151 | 0.903 | 0.594 | 6.084E-97 | B_Mem_Early-Ccr6 | Ifi30 |
| 62 | 2.180E-91 | 1.325899537 | 0.666 | 0.285 | 5.495E-87 | B_Mem_Early-Ccr6 | Ccr6 |
| 63 | 1.064E-55 | 0.83828031 | 0.773 | 0.467 | 2.681E-51 | B_Mem_Early-Ccr6 | Cd72 |
| 64 | 4.783E-55 | 1.202186854 | 0.56 | 0.278 | 1.205E-50 | B_Mem_Early-Ccr6 | Bcl2a1b |
| 65 | 5.935E-52 | 0.793759515 | 0.81 | 0.537 | 1.496E-47 | B_Mem_Early-Ccr6 | Gimap4 |
| 66 | 6.116E-48 | 0.811747974 | 0.719 | 0.431 | 1.541E-43 | B_Mem_Early-Ccr6 | Cd38 |
| 67 | 8.826E-43 | 0.776340998 | 0.797 | 0.559 | 2.224E-38 | B_Mem_Early-Ccr6 | Cotl1 |
| 68 | 2.119E-41 | 0.82696488 | 0.685 | 0.432 | 5.339E-37 | B_Mem_Early-Ccr6 | Arhgap24 |
| 69 | 2.727E-41 | 1.003060376 | 0.569 | 0.318 | 6.873E-37 | B_Mem_Early-Ccr6 | Gngt2 |
| 70 | 2.163E-39 | 0.849797726 | 0.61 | 0.354 | 5.452E-35 | B_Mem_Early-Ccr6 | H2-Eb2 |
| 71 | 6.162E-34 | 1.259176087 | 0.293 | 0.119 | 1.553E-29 | B_Mem_Early-Ccr6 | Bcl2a1d |
| 72 | 9.282E-34 | 0.789737557 | 0.68 | 0.463 | 2.339E-29 | B_Mem_Early-Ccr6 | Mrpl52 |
| 73 | 1.074E-32 | 0.865755455 | 0.597 | 0.373 | 2.706E-28 | B_Mem_Early-Ccr6 | Blvrb |
| 74 | 4.869E-31 | 0.877853591 | 0.521 | 0.295 | 1.227E-26 | B_Mem_Early-Ccr6 | Cr2 |
| 75 | 4.635E-30 | 0.847100044 | 0.444 | 0.23 | 1.168E-25 | B_Mem_Early-Ccr6 | Pxdc1 |
| 76 | 1.904E-23 | 0.825515286 | 0.519 | 0.337 | 4.797E-19 | B_Mem_Early-Ccr6 | Ifngr2 |
| 77 | 4.510E-21 | 0.776642676 | 0.417 | 0.247 | 1.136E-16 | B_Mem_Early-Ccr6 | AW112010 |
| 78 | 3.263E-19 | 0.783679652 | 0.363 | 0.208 | 8.222E-15 | B_Mem_Early-Ccr6 | Cyfip1 |
| 79 | 7.540E-19 | 0.825116024 | 0.348 | 0.198 | 1.900E-14 | B_Mem_Early-Ccr6 | Bhlhe40 |
| 80 | 2.651E-17 | 0.794335559 | 0.356 | 0.211 | 6.680E-13 | B_Mem_Early-Ccr6 | Rab30 |
| 81 | 9.746E-247 | 5.410486295 | 0.539 | 0.018 | 2.456E-242 | B_Mem_Core-Cd80 | Cxcr3 |
| 82 | 1.528E-227 | 4.892018334 | 0.681 | 0.039 | 3.850E-223 | B_Mem_Core-Cd80 | Cd80 |
| 83 | 6.665E-218 | 5.49620941 | 0.496 | 0.018 | 1.680E-213 | B_Mem_Core-Cd80 | Trerf1 |
| 84 | 1.749E-171 | 4.894016604 | 0.418 | 0.016 | 4.408E-167 | B_Mem_Core-Cd80 | Zbtb32 |
| 85 | 5.327E-163 | 5.598403719 | 0.305 | 0.007 | 1.342E-158 | B_Mem_Core-Cd80 | Tnfrsf8 |
| 86 | 4.115E-134 | 4.614074439 | 0.312 | 0.011 | 1.037E-129 | B_Mem_Core-Cd80 | Trabd2b |
| 87 | 7.436E-120 | 4.0177527 | 0.504 | 0.043 | 1.874E-115 | B_Mem_Core-Cd80 | Sspn |
| 88 | 4.914E-118 | 3.199571662 | 0.688 | 0.084 | 1.238E-113 | B_Mem_Core-Cd80 | Scimp |
| 89 | 5.266E-108 | 3.765203105 | 0.44 | 0.035 | 1.327E-103 | B_Mem_Core-Cd80 | Nt5e |
| 90 | 6.212E-107 | 3.532959363 | 0.511 | 0.05 | 1.565E-102 | B_Mem_Core-Cd80 | Bhlhe41 |
| 91 | 8.665E-105 | 3.01440953 | 0.56 | 0.06 | 2.183E-100 | B_Mem_Core-Cd80 | Myo1f |
| 92 | 6.925E-93 | 3.031383839 | 0.567 | 0.071 | 1.745E-88 | B_Mem_Core-Cd80 | Ass1 |
| 93 | 8.444E-91 | 4.726504334 | 0.397 | 0.036 | 2.128E-86 | B_Mem_Core-Cd80 | Tox |
| 94 | 2.539E-85 | 2.804788585 | 0.574 | 0.079 | 6.398E-81 | B_Mem_Core-Cd80 | Fas |
| 95 | 4.352E-84 | 4.545681306 | 0.27 | 0.016 | 1.097E-79 | B_Mem_Core-Cd80 | Bmpr1a |
| 96 | 1.687E-63 | 2.858681932 | 0.369 | 0.043 | 4.252E-59 | B_Mem_Core-Cd80 | Lacc1 |
| 97 | 1.615E-60 | 3.60353879 | 0.27 | 0.024 | 4.070E-56 | B_Mem_Core-Cd80 | Plcd3 |
| 98 | 2.019E-44 | 2.872530692 | 0.39 | 0.069 | 5.088E-40 | B_Mem_Core-Cd80 | Cacna1d |
| 99 | 3.903E-40 | 3.283718512 | 0.397 | 0.077 | 9.836E-36 | B_Mem_Core-Cd80 | Rgs1 |
| 100 | 3.587E-36 | 2.905555375 | 0.284 | 0.045 | 9.039E-32 | B_Mem_Core-Cd80 | Ifi211 |
| 101 | 1.718E-140 | 0.538408833 | 1 | 0.985 | 4.330E-136 | B_EarlyAct-Junb | Rps27 |
| 102 | 4.143E-77 | 0.507782134 | 0.998 | 0.99 | 1.044E-72 | B_EarlyAct-Junb | Fth1 |
| 103 | 3.331E-73 | 0.663291375 | 0.986 | 0.955 | 8.395E-69 | B_EarlyAct-Junb | Junb |
| 104 | 1.066E-43 | 0.568315972 | 0.897 | 0.782 | 2.686E-39 | B_EarlyAct-Junb | Cd55 |
| 105 | 1.598E-40 | 0.660938107 | 0.836 | 0.76 | 4.027E-36 | B_EarlyAct-Junb | Ier2 |
| 106 | 1.634E-39 | 0.503797145 | 0.935 | 0.809 | 4.117E-35 | B_EarlyAct-Junb | Ighd |
| 107 | 4.814E-31 | 0.53843745 | 0.787 | 0.768 | 1.213E-26 | B_EarlyAct-Junb | Rps27rt |
| 108 | 1.149E-30 | 0.592779224 | 0.774 | 0.72 | 2.894E-26 | B_EarlyAct-Junb | Gmfg |
| 109 | 7.526E-27 | 0.495834844 | 0.773 | 0.649 | 1.897E-22 | B_EarlyAct-Junb | Sell |
| 110 | 3.041E-22 | 0.530221123 | 0.754 | 0.723 | 7.662E-18 | B_EarlyAct-Junb | D8Ertd738e |
| 111 | 3.356E-14 | 0.526877673 | 0.593 | 0.572 | 8.457E-10 | B_EarlyAct-Junb | Ptp4a3 |
| 112 | 3.858E-14 | 0.747820034 | 0.31 | 0.212 | 9.722E-10 | B_EarlyAct-Junb | Pde2a |
| 113 | 8.781E-13 | 0.520758496 | 0.577 | 0.559 | 2.213E-08 | B_EarlyAct-Junb | Ypel3 |
| 114 | 4.396E-12 | 0.539849899 | 0.546 | 0.506 | 1.108E-07 | B_EarlyAct-Junb | Rasgrp2 |
| 115 | 8.265E-12 | 0.590038284 | 0.604 | 0.591 | 2.083E-07 | B_EarlyAct-Junb | Cebpb |
| 116 | 1.682E-10 | 0.632561932 | 0.443 | 0.4 | 4.239E-06 | B_EarlyAct-Junb | Cirbp |
| 117 | 3.302E-09 | 0.505812145 | 0.722 | 0.71 | 8.320E-05 | B_EarlyAct-Junb | S100a9 |
| 118 | 3.307E-08 | 0.490425138 | 0.561 | 0.575 | 8.333E-04 | B_EarlyAct-Junb | Ppdpf |
| 119 | 7.946E-08 | 0.542720782 | 0.445 | 0.429 | 2.002E-03 | B_EarlyAct-Junb | Tuba1a |
| 120 | 1.060E-07 | 0.689804378 | 0.419 | 0.386 | 2.671E-03 | B_EarlyAct-Junb | Wfdc17 |
| 121 | 2.335E-76 | 3.479318336 | 0.578 | 0.078 | 5.884E-72 | B_Prolif-Ccnd2 | Srm |
| 122 | 3.944E-76 | 3.014607527 | 0.908 | 0.24 | 9.938E-72 | B_Prolif-Ccnd2 | Ppp1r14b |
| 123 | 5.136E-66 | 2.981698644 | 0.624 | 0.103 | 1.294E-61 | B_Prolif-Ccnd2 | Timm8a1 |
| 124 | 4.306E-63 | 2.712960476 | 0.872 | 0.243 | 1.085E-58 | B_Prolif-Ccnd2 | Nhp2 |
| 125 | 3.685E-55 | 2.56227997 | 0.89 | 0.288 | 9.285E-51 | B_Prolif-Ccnd2 | Pa2g4 |
| 126 | 2.504E-52 | 2.779651721 | 0.541 | 0.093 | 6.310E-48 | B_Prolif-Ccnd2 | Apex1 |
| 127 | 3.365E-51 | 3.054576928 | 0.541 | 0.099 | 8.480E-47 | B_Prolif-Ccnd2 | Mettl1 |
| 128 | 4.549E-51 | 2.63275249 | 0.651 | 0.143 | 1.146E-46 | B_Prolif-Ccnd2 | Mrto4 |
| 129 | 3.927E-48 | 3.257395596 | 0.275 | 0.026 | 9.896E-44 | B_Prolif-Ccnd2 | Shmt1 |
| 130 | 4.063E-47 | 2.615816969 | 0.495 | 0.084 | 1.024E-42 | B_Prolif-Ccnd2 | Atad3a |
| 131 | 1.238E-45 | 2.90188802 | 0.33 | 0.039 | 3.119E-41 | B_Prolif-Ccnd2 | Gcsh |
| 132 | 9.544E-45 | 2.741571766 | 0.468 | 0.081 | 2.405E-40 | B_Prolif-Ccnd2 | Snhg4 |
| 133 | 3.228E-44 | 2.703555827 | 0.872 | 0.331 | 8.134E-40 | B_Prolif-Ccnd2 | Ccnd2 |
| 134 | 3.090E-43 | 2.563883516 | 0.505 | 0.096 | 7.787E-39 | B_Prolif-Ccnd2 | Dctpp1 |
| 135 | 2.420E-39 | 2.972834906 | 0.294 | 0.036 | 6.098E-35 | B_Prolif-Ccnd2 | Trmt61a |
| 136 | 2.543E-36 | 2.673215002 | 0.431 | 0.084 | 6.407E-32 | B_Prolif-Ccnd2 | Lap3 |
| 137 | 7.042E-36 | 2.936795345 | 0.339 | 0.054 | 1.775E-31 | B_Prolif-Ccnd2 | Hk2 |
| 138 | 3.825E-35 | 2.755863481 | 0.404 | 0.076 | 9.638E-31 | B_Prolif-Ccnd2 | Nop16 |
| 139 | 3.162E-31 | 2.562835033 | 0.284 | 0.041 | 7.967E-27 | B_Prolif-Ccnd2 | Cdca7 |
| 140 | 1.086E-28 | 2.60984079 | 0.44 | 0.104 | 2.737E-24 | B_Prolif-Ccnd2 | Il4i1 |
| 141 | 0.000E+00 | 7.846712691 | 0.933 | 0.011 | 0.000E+00 | B_Plasma | Derl3 |
| 142 | 0.000E+00 | 10.20643538 | 0.996 | 0.103 | 0.000E+00 | B_Plasma | Jchain |
| 143 | 0.000E+00 | 8.615757504 | 0.893 | 0.029 | 0.000E+00 | B_Plasma | Iglv1 |
| 144 | 0.000E+00 | 9.155688298 | 0.83 | 0.005 | 0.000E+00 | B_Plasma | Tnfrsf17 |
| 145 | 0.000E+00 | 6.215316177 | 0.807 | 0.02 | 0.000E+00 | B_Plasma | Osbpl3 |
| 146 | 0.000E+00 | 6.447880434 | 0.774 | 0.016 | 0.000E+00 | B_Plasma | Cd28 |
| 147 | 0.000E+00 | 6.963133472 | 0.689 | 0.011 | 0.000E+00 | B_Plasma | Dnm3 |
| 148 | 0.000E+00 | 10.59583585 | 0.589 | 0.001 | 0.000E+00 | B_Plasma | Ccr10 |
| 149 | 0.000E+00 | 6.882356601 | 0.585 | 0.004 | 0.000E+00 | B_Plasma | Nacc2 |
| 150 | 0.000E+00 | 7.286862358 | 0.481 | 0.002 | 0.000E+00 | B_Plasma | Hid1 |
| 151 | 0.000E+00 | 7.347824615 | 0.459 | 0.001 | 0.000E+00 | B_Plasma | Bhlha15 |
| 152 | 8.751E-275 | 6.197838272 | 0.404 | 0.009 | 2.205E-270 | B_Plasma | Cped1 |
| 153 | 1.043E-247 | 9.405181973 | 0.274 | 0 | 2.629E-243 | B_Plasma | Prg2 |
| 154 | 7.820E-244 | 6.936317316 | 0.281 | 0.001 | 1.971E-239 | B_Plasma | Fads2 |
| 155 | 6.974E-241 | 8.407092521 | 0.263 | 0 | 1.757E-236 | B_Plasma | Tspan15 |
| 156 | 6.279E-234 | 8.242040217 | 0.256 | 0 | 1.582E-229 | B_Plasma | Prss57 |
| 157 | 5.849E-229 | 6.750142796 | 0.285 | 0.003 | 1.474E-224 | B_Plasma | Oosp1 |
| 158 | 2.398E-177 | 9.547898599 | 0.952 | 0.321 | 6.043E-173 | B_Plasma | Igha |
| 159 | 1.656E-121 | 6.713290107 | 0.663 | 0.14 | 4.173E-117 | B_Plasma | Slpi |
| 160 | 1.072E-68 | 7.962980515 | 0.333 | 0.055 | 2.702E-64 | B_Plasma | Ighg2b |

**Supplementary Table S5.** Demographic and clinical characteristics of pediatric patients with HAdV infection and healthy controls.

| **Characteristic** | **Control (n = 9)** | **HAdV(n=13)** | ***P_*value** |
| --- | --- | --- | --- |
| **Male sex, n (%)** | 4 (44.4) | 9 (69.2) | 0.3842 |
| **Age, years, median (IQR)** | 9.1 (5.3–9.4) | 1.3 (0.9–3.8) | 0.0026 |
| **Severe disease, n (%)** | — | 11 (84.6) | — |
| **ICU admission, n (%)** | — | 5 (38.5) | — |
| **Therapy before sampling, n (%)** |  |  |  |
| Antibiotics | — | 13 (100) | — |
| Antiviral treatment | — | 0 (0) | — |
| Corticosteroids | — | 5 (38.5) | — |
| IVIG | — | 1 (7.7) | — |
| Non-invasive respiratory support | — | 2 (15.4) | — |
| Invasive mechanical ventilation | — | 0 (0) | — |
| **Hospital stay, days, median (IQR)** | — | 10 (6–14) | — |
| **Discharged, n (%)** | — | 13 (100) | — |

ICU, intensive care unit; IVIG, intravenous immunoglobulin; —, not applicable.
